# Social factors influencing utilization of home care in community-dwelling older adults: a scoping review

**DOI:** 10.1186/s12877-021-02069-1

**Published:** 2021-02-27

**Authors:** Jasmine C. Mah, Susan J. Stevens, Janice M. Keefe, Kenneth Rockwood, Melissa K. Andrew

**Affiliations:** 1 Department of Health Policy, London School of Economics and Political Sciences, London, UK; 2 Faculty of Public Health and Policy, London School of Hygiene and Tropical Medicine, London, UK; 3 Department of Medicine, Dalhousie University, Halifax, NS Canada; 4 Faculty of Family Studies and Gerontology, Mount Saint Vincent University, Halifax, NS Canada; 5 Continuing Care, Nova Scotia Health, Halifax, Nova Scotia Canada; 6 Division of Geriatric Medicine, Dalhousie University, Halifax, NS Canada

**Keywords:** Health care utilization, Home health care, Community care, Influencing aspects, Formal care, Social support, Social vulnerability

## Abstract

**Background:**

Older adults want to live at home as long as possible, even in the face of circumstances that limit their autonomy. Home care services reflect this emergent preference, allowing older adults to ‘age in place’ in familiar settings rather than receiving care for chronic health conditions or ageing needs in an institutionalized setting. Numerous social factors, generally studied in isolation, have been associated with home care utilization. Even so, social circumstances are complex and how these factors collectively influence home care use patterns remains unclear.

**Objectives:**

To provide a broad and comprehensive overview of the social factors influencing home care utilization; and to evaluate the influence of discrete social factors on patterns of home care utilization in community-dwelling older adults in high-income countries.

**Methods:**

A scoping review was conducted of six electronic databases for records published between 2010 and 2020; additional records were obtained from hand searching review articles, reference lists of included studies and documents from international organisations. A narrative synthesis was presented, complemented by vote counting per social factor, harvest plots and an evaluation of aggregated findings to determine consistency across studies.

**Results:**

A total of 2,365 records were identified, of which 66 met inclusion criteria. There were 35 discrete social factors grouped into four levels of influence using a socio-ecological model (individual, relationship, community and societal levels) and grouped according to outcome of interest (home care propensity and intensity). Across all studies, social factors consistently showing any association (positive, negative, or equivocal in pattern) with home care propensity were: age, ethnicity/race, self-assessed health, insurance, housing ownership, housing problems, marital status, household income, children, informal caregiving, social networks and urban/rural area. Age, education, personal finances, living arrangements and housing ownership were associated with home care intensity, also with variable patterns in utilization. Additional community and societal level factors were identified as relevant but lacking consistency across the literature; these included rurality, availability of community services, methods of financing home care systems, and cultural determinants.

**Conclusion:**

This is the first literature review bringing together a wide range of reported social factors that influence home care utilization. It confirms social factors do influence home care utilization in complex interactions, distinguishes level of influences at which these factors affect patterns of use and discusses policy implications for home care reform.

**Supplementary Information:**

The online version contains supplementary material available at 10.1186/s12877-021-02069-1.

## Background

In the face of global population ageing and individuals living longer with chronic health conditions, governments, healthcare advocates and older adults themselves are tasked with finding the balance between rising healthcare expenditures and improving older people’s health and wellbeing. Many countries have opted to shift the site of care provision from hospitals and long-term care facilities to home and community settings, with the “expectation of cost savings, or at the very least, that such substitution might lead to more efficient use of nursing home and hospital beds” [1, 2] (p.108). The World Health Organization’s Global Strategy and Action Plan on Ageing and Health also highlights the importance of delivering home and community-based care to enable older people to 'age in place' with dignity [3, 4]. As a result, home care is becoming a national priority in many countries.

There is no universal definition of home care. This paper defines home care as “services [that] help people to receive care at home, rather than in a hospital or long-term care facility, and to live as independently as possible in the community.” [5] The focus of this paper is on formal (paid) home care rather than informal home care (unpaid care provided by family, friends or neighbours). Formal home care encompasses both home health care (HHC) services and home support services (HSS) [6]. The former refers to nursing or rehabilitative care and other services delivered by licensed health professionals. The latter refers to personal care, housework, meal preparation, and/or respite care delivered by personal support workers or volunteer agencies. The services encompassed under the term home care are often arbitrary. Similarly, depending on context, home care is often characterized under broader services in the healthcare and social care sectors such a long-term care services, community aged-care services, etc. Nevertheless, the goals of all services are to maintain or improve quality of life and functional abilities to promote greater independence and satisfaction while living at home or in the community.

The emphasis on home care reflects an emergent widespread preference; older adults who require assistance prefer to 'age in place' in familiar settings rather than receive care for dementia, other chronic health conditions or ageing needs in an institutionalized setting [7–10]. For the individual, home care has been associated with decreased mortality, reduced hospitalisations, delayed institutionalisation and improved quality of life [11–13]. At a health system level, in comparison to long-term care facilities or hospitalisations, home care in the community has been associated with significantly lower overall healthcare costs [14]. The appeal of home care is that it can produce health outcomes comparable to those achieved in institutionalised settings, respond to the call for personalised care for individuals to live in the comfort of their own home and improve cost-efficiency [1].

For home care to serve the above purposes, it is important to establish the characteristics of the individuals who use home care services. This provides “the fundamental information necessary to develop the most cost-effective services for each patient group… This information will also allow healthcare providers and policymakers to prepare and provide services” for patients [15](p.512). As the population ages, governments and policymakers need to know the current drivers of service use, which will allow them to predict how to best allocate resources and manage the expected increase in demand on the home care system [16]. Significant modifications and reforms to the home care system become more feasible when we understand what drives utilization.

Unfortunately, the predictors of home care utilization are unevenly understood; while the health determinants impacting home care use are well established by previous reviews [17, 18], there is less literature looking at how social factors collectively impact use of home care services. Health factors such as cognitive impairment, mobility issues and number of chronic conditions (collectively known as frailty) frequently increase use of home care services [15, 17, 19]; this trend is in keeping with general patterns that healthcare services, not specifically home care, are positively correlated with frailty [20, 21]. But the nature of home care service provision relies on resources in the community, beyond individual health status. When two individuals have the same degree of health problems, the difference between being able to remain independent at home with assistance and having to find alternate living accommodations can be explained by social circumstances [22].

Social factors (or social determinants, terms are used interchangeably in this paper) are defined as the conditions in which people live, work and age, influencing their health and care needs [23]. Though potentially relevant for people of all ages, the protective or deleterious accumulation of social factors is particularly relevant in older age [22, 24]. Older adults using home care services are increasingly reliant on social supports (i.e. caregivers and community resources), but at the same time, have dwindling social networks (due to death of friends and family, or greater difficulty participating in social activities due to health and functional impairments) [22]. And while there have been studies investigating social determinants that affect home care use, most fail to look at the whole picture by focusing on a single or a limited number of determinants [22]. Therefore, drawing upon a socio-ecological framework, the overarching purpose of this paper is to provide a broad and comprehensive overview of all the social factors influencing home care utilization, which is a current gap in the literature; to our knowledge, a review bringing together the whole range of social determinants has not previously been conducted.

## Methods

### Objectives

1. To provide (by identifying and describing) an overview of reported social factors influencing home care utilization, exploring the breadth (comprehensiveness), rather than depth (details) of available evidence to answer the research question.
2. To evaluate the influence of each discrete social factor on patterns of home care utilization.

### Search strategy

A scoping review was conducted using the Arksey and O’Malley (2005) framework refined by Levac, Colquohoun and O’Brien (2010) [25, 26]. A search of primary studies was conducted in MEDLINE, EMBASE, SCOPUS, Social Science Citation Index (SSCI), the National Health Service Economic Evaluation Database (NHS EED) and Cochrane Library. The last search was conducted July 12, 2020. The search was developed in MEDLINE after consultation with a health sciences librarian, and then translated to the subsequent databases (full search available in Additional File 1). To ensure the findings of the review were relevant to social circumstances in high-income countries, a validated low-and-middle-income country (LMIC) search filter was applied to exclude studies conducted in these countries as defined by the World Bank [27]. Additionally, documents from the websites of two international bodies (World Health Organisation’s Ageing and Life Course section and the International Home Care Nurses Organisation) and reference lists of included studies and previous literature reviews were hand-searched for records [28, 29].

Citations were imported into Covidence (2020), a systematic review web platform that removes duplicates and facilitates screening of titles and abstracts, full text retrieval and eligibility assessment [30]. The first author was responsible for screening of titles and abstracts using Covidence. She also retrieved and screened the full-text articles using the same platform. All authors contributed to establishing the scoping review protocol at the beginning of the study and were consulted throughout the process of data screening and retrieval to ensure adherence to the planned review protocol. The scoping review protocol was not registered but this paper does follow PRISMA-ScR guidelines (see Additional File 2).

### Eligibility criteria

Studies were included when:

- Study participants were community-dwelling older adults greater or equal to 60 years of age living in high-income countries (population). Studies were included if > 50% of the sample met this criteria.
- Studies examined a social factor (intervention or exposure) in relation to home care services. Social factors included in this review had to meet the definition of at least one social determinant of health (income and social status, employment and working conditions, education and literacy, childhood experience, physical environments, social supports and coping skills, healthy behaviours, access to health services, biology and genetic endowment, gender, culture, race/racism) [23, 31].
- Study findings included the use of formal home care services (outcome). Utilization (or use) refers to whether clients have actually received home care services. Therefore, need and access are not outcomes included in this review. Informed by previous literature, outcomes domains of home care service utilization can be divided into:
  - 1) Intensity of home care use - defined as the amount of services (hours of service, number of services, costs of home care, etc.); and
  - 2) Propensity home care use – defined as a dichotomized outcome of having received or not received any home care [1, 32].
- Studies were quantitative (type of study); quantitative studies were felt by the authors to better answer the second objective of the scoping review (to evaluate the discrete influence of each social factor on propensity and intensity of home care use), although we acknowledge that qualitative studies have the potential to add a richness to the understanding of the issue and thus consideration of results from qualitative literature represents an important area for further exploration.

Non-English studies and studies prior to 2010 were excluded. The authors feel that the approach to caring for ageing populations in their own home is best reflected in more recent literature. By limiting the search to the last ten years, this scoping review enables assessment of relevant practices and challenges in home care, which may have more policy relevance in the current political and healthcare climate. Studies looking primarily at long-term care homes or hospital based care programs (i.e. day hospital programs, which can be considered part of home care in some countries) were excluded. Studies focusing on populations of older adults with dementia or diagnosed palliative conditions were also excluded; the former due to a recent scoping review in patients with dementia and the latter because a palliative population often has access to additional care services [18].

### The socio-ecological model

The complex, interconnected relationships between individuals, their social circumstances and their environment present a challenge for researchers who study social determinants of health [33]. In particular, the patterns of how social circumstances affect health behaviours are not easily explained by considering each social factor in isolation. To illustrate, educational attainment is a property of individuals; yet, ability to go to school is associated with household income and both are measures of neighbourhood deprivation and unemployment. The socio-ecological model provides a way of conceptualizing and disentangling this complex interplay while still acknowledging the contribution of all factors. Based on ecological systems theory by Bronfenbrenner, the model assumes that health behaviours are shaped by the individual’s relationships with caregivers (micro and meso systems) the community (exosystem) and broader society (macrosystem) as opposed to health behaviours solely being the product of individual characteristics and choices [34].

In applying the socio-ecological model to this review, it is therefore assumed that home care use (the health behaviour) is influenced by factors at each level of influence to various degrees. The social factors identified by this review will be organised into four levels of influence: individual (microsystem), relationship (meso-system), community (exosystem) and societal (macrosystem). The individual level refers to the intrapersonal characteristics of the individual who requires home care. The relationship level examines the interpersonal relationship between the individual and their caregivers. The community level explores the assets or deficits in the community that impact home care. The societal level refers to the public policy and cultural norms that shape the availability of home care services. These factors impacting home care utilization interact across all levels to impact behaviour change. The social-ecological model is best understood as a dynamic model for the purposes of this review, allowing for factors to be added or removed from categories of influence (e.g. household income can be a relationship factor for married adults, but an individual factor for single older adults).

### Data Extraction & Analysis

A data extraction form was created to collect information from each paper. This included: (1) General information (country and type of home care system, ethics, funding sources, conflicts of interest); (2) Study methods (aim, design, start/end dates, data source, model/theory); (3) Participants (inclusion/exclusion criteria, method of recruitment, characteristics); (4) Social factors and exposures; (5) Outcome data (relevant to social factors); (6) Key author conclusions relevant to the review question.

The high heterogeneity of study populations, designs, data sources and statistical adjustment techniques (in addition to the variable ways of measuring social factors and home care outcomes and inconsistent types of effect measures) precluded a statistical combination of results through meta-analytic techniques. Instead, in line with the scoping review framework, the following process of collating, summarizing and reporting occurred to produce a narrative rather than statistical summary.

First, all studies were investigated using a qualitative content analysis approach including reduction, explication and restructuring [18]. After the content from each study was reduced to only the social determinants influencing home care, the material was organised in relation to the socio-ecological model and grouped according to outcome (intensity or propensity). Since multiple studies provided results for more than one social factor, studies may appear in multiple areas of the framework. To complement the narrative synthesis, vote counting per social factor occurred based on direction of effect and statistical significance. Vote counting synthesizes information to answer the question: is there any evidence of effect? This question is particularly relevant for the objectives of this scoping review [35]. Where appropriate (for at least 2 studies per social factor), harvest plots were created to visualize vote counting per social factor and outcome domains, accounting for study type (colour) and study measures (labelled on x-axis) [35–37]. Harvest plots group studies based on direction of effect (i.e. positive association, null association, negative association) and each study is represented by a bar positioned according to its categorization [35].

Second, an evaluation of aggregated findings per social factor was conducted to determine consistency across findings. This evaluation process has been endorsed by previous narrative reviews on healthcare utilization [17, 21, 38].

- Step 1: If the majority (≥60%) of the studies indicated that a social factor was associated (either positively or negatively) with home care utilization, then that social factor was evaluated as likely “associated” across all studies. If 40–59% of the studies showed an association (either positively or negatively), then the outcome of the evaluation for this social factor was deemed “uncertain”. If < 40% of the studies supported any association (either positively or negatively), the consensus is that the social factor is likely “not associated” with home care utilization across all studies.
- Step 2: For social factors that were deemed “associated” in Step 1, the same evaluation took place using all significant studies (positively and negatively associated) as the new denominator. The same cut offs above were used to determine the direction/pattern of the association: “positive/more”, “negative/less”, or “equivocal”.
- The evaluation was only performed for social factors with at least 2 studies. If studies reported both univariate and multivariate analyses, only the multivariate results were included.

All simple statistics (i.e. frequencies, proportions) were conducted with STATA-IC 16.1. All figures were created using Microsoft Excel or Lucidchart [39].

## Results

### Description of studies

**Fig. 1:**
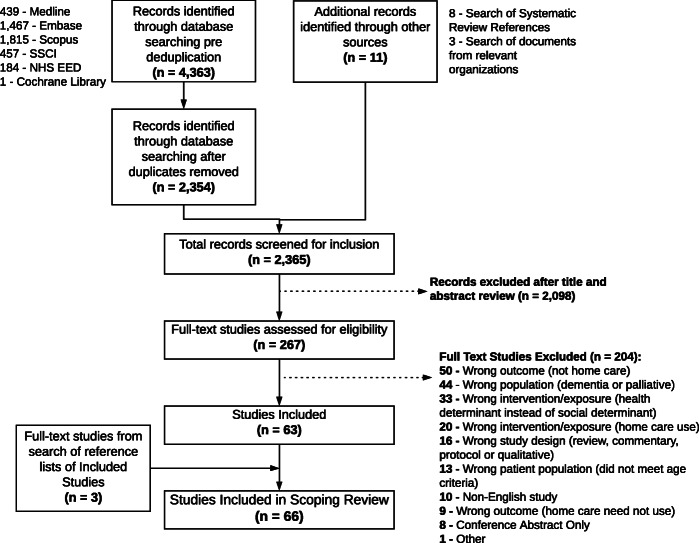
Flowchart of study selection

The electronic database search identified 2,354 unique records. An additional 11 records were identified by other means (See Fig. 1 for details). After title and abstract screening, 267 studies met the inclusion criteria and underwent full-text review. With the addition of three studies identified from hand-searching the references of included studies, a total of 66 studies were included in this scoping review for analysis [40–105].

#### Study characteristics

Most of the studies (44 studies or 66.7%) were secondary data analyses of existing databases, [42, 44, 45, 47, 50, 51, 54, 57, 59–63, 65, 66, 68, 69, 71, 72, 74–76, 80, 82, 83, 87, 90, 93–96, 100–102]; of which, ten of these studies used econometric analytic techniques [41, 43, 56, 64, 77, 84, 86, 91, 97, 98]. Other study designs included nine cross-sectional surveys [40, 48, 55, 58, 79, 81, 85, 103, 105], five longitudinal cohort studies [46, 49, 70, 78, 88], four retrospective cohort studies [73, 89, 99, 104], and one each of a case control [53], quasi experimental [52], cluster randomized controlled trial [92] and quality improvement study [67]. Fifty-six studies (84.9%) controlled for health status in some way, either in study design or using statistical techniques. Probabilities of home care utilization were most commonly estimated using logistic regression (30 studies or 45.5%).

The majority of studies did not reflect the influence of a theory or model (34 studies or 51.5%). The most common model used to study home care utilization was Andersen’s behavioural model in 24 studies (36.4%). Additional theories and models were: cumulative advantage/disadvantage theory, Lawton’s ecological model, Person-Environment fit theories, Grossman’s model of demand for health, network-episode model and Becker’s model of family decision making.

These studies were based on 64 unique data sources, after accounting for studies that linked multiple datasets. The Survey of Health, Ageing and Retirement in Europe (SHARE) was the most common analysed dataset in seven studies [41, 43, 54, 63, 68, 82, 84]. Additional information summarizing the data sources by country is available in Additional File 3. Participants were included from 23 countries, primarily from the United States of America (USA) (21 studies or 31.8%). Sample sizes ranged from 214 to 41,431,788 participants. Mean and median sample sizes were 721,781 participants and 6,551 participants, respectively. There was high heterogeneity in populations sampled, including a wide range of functional impairments, frailty scores, diagnosed medical conditions and social characteristics. Some studies used insurance claims or enrolment to identify their populations, while others used national census databases, medical clinical enrolment, or hospital discharge databases. The key characteristics of all 66 studies are summarized table form in Additional File 4.

**Fig. 2:**
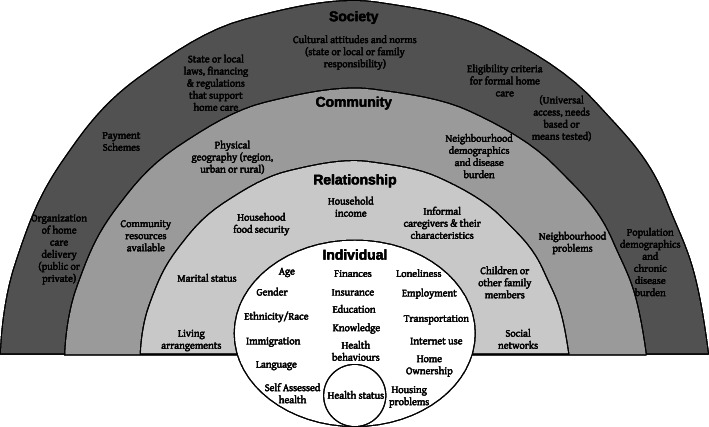
A socio-ecological model for social factors influencing utilization of home care in community-dwelling older adults. This model is meant to be dynamic in nature; factors can be added, removed, or moved to different levels of influence to suit the context

The review identified 17 unique social determinants influencing home care at an individual level, nine factors at the relationship level, five at the community level and four factors at the society level. Guided by the generic socio-ecological model [34], all findings from this scoping review have been arranged into a socio-ecological model as it applies to social factors and home care, depicted in Fig. 2.

Home care propensity was examined in 57 studies (86.4%) and 18 studies examined home care intensity (27.3%). Only ten studies further separated home care into divisions that resemble home health care and home support services (15.2%) [60, 65, 67, 76, 79, 83, 88, 94, 104, 105]. The terminology and definition of home care slightly varied depending on study; alternate designations for home care are indicated in Table 1.

**Table 1:** List of terms identified in the included studies that designated services equivalent to home care

Community aged care packages (CACP)	
Community-based long-term care (CBLTC)	
Formal support	
Home and community based services (HCBS)	
Home and community care (HACC)	
Home care packages (HCP)	
Home care services (HCS)	
Home health aides (HHA) services	
Home health agency admissions	
Home health care (HHC)	
Home help services (HHS)	
Long-term care (LTC)	
Long-term care services (LTCS)	
Skilled home health visits	
Skilled nursing visits	

### Social factors influencing home care

#### Level: individual

##### Age

**Fig. 3:**
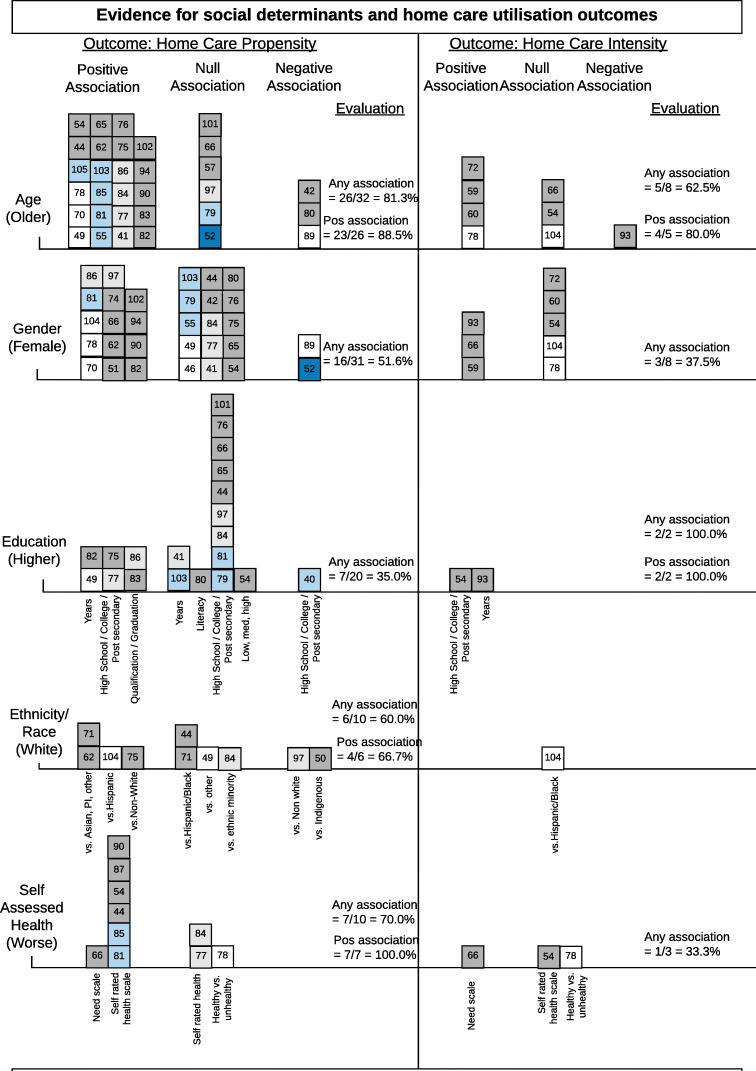
Harvest plot summarizing consistency of evidence for individual level social determinants associated with home care propensity and intensity. Each social determinant is labelled on the left and each box represents one study. The number in the box corresponds to the study number in the references section. The social determinants are arranged in descending order based on counts of studies. The colour of the box indicates the study design (white = cohort, dark grey = secondary data analysis, light grey = secondary econometric analysis, dark blue = experimental, light blue = cross-sectional, pink = quality improvement). Where relevant, the x-axis is labelled with measures used to define the social determinant or to indicate a comparison group (denoted by vs.). The evaluation of aggregated findings is also summarized numerically in the right columns

Age was the most frequently examined factor or covariate in 32 studies. The influence of age on the propensity and intensity of home care was evaluated as “associated”, that is to say there was a consistent association across all studies in the review. Figure 3 shows that 81.3% of the studies looking at age and home care propensity showed a statistically significant relationship and of those, 88.5% showed that older age is positively associated with more home care. This can be explained because increasing age is associated with higher frailty and chronic health conditions but also shrinking social support networks. Of note, one study from the Netherlands found that home care use increases with age until a certain point, then the probability of returning home with home care declined and the probability of institutionalized care increased; this age of transition was found to be 90 years old [102].

##### Gender

Gender is often recognized as a social determinant that compounds the disparities in income, wealth and education, particularly over time, which in turn influences healthcare use. However, across 31 studies for home care propensity and 8 studies for home care intensity, gender was evaluated to have an uncertain relationship on home care utilization. While fourteen studies found women used more homes care services than men [51, 62, 66, 70, 74, 78, 81, 82, 86, 90, 94, 97, 102, 104], an equal number of studies did not show a significant difference in service use [41, 42, 44, 46, 49, 54, 55, 65, 75–77, 79, 80, 84, 103].

For studies supporting higher propensity among women, women had a higher probability of first-time use of any services, were more likely to seek out home care services and higher probability of using support services [74, 93, 94]. Another study suggested that increased home care use can be explained by older age in women recipients and being widowed having married older men. While men have a higher probability of receiving care from a co-habiting partner, women were more likely to report their caregivers are outside of the household, mainly children or community services [74]. This may help explain why women were more likely to be users of hospice care and long-term institutional care [51, 74, 102]. Cameron and colleagues initially found female predominance in home care use, but the relationship was attenuated after adjusting for living arrangements, health needs and economic access factors [46]. Interestingly, while the odds of women using home care services are higher, they may not have higher service needs or costs compared to men [52, 60, 104]. Most studies did not break down home care services into health services and personal support services but the breakdown of services types used by women compared to men would be helpful in understanding these gender differences.

##### Education

Level of education was not associated with home care propensity. Higher education was, however, associated with increased home care intensity. Deindl and Brandt (2017) found that highly educated older adults received more hours per week of formal and combined (formal and informal) home care compared to those with low or medium education [54]; and Shih and colleagues (2020) found a positive association between years of education and length of time in the home care system [93]. Figure 3 shows that the measures of education between studies varied greatly (e.g. measuring education by literacy and illiteracy in Taiwan [80] is likely to produce different outcomes than measuring education by post-secondary and secondary school in Canada [40]), contributing to the differences in influence on home care outcomes.

##### Ethnicity/race

Ethnicity/race was evaluated as borderline associated with home care propensity (60%). In four of six studies with any association between this social determinant and home care propensity, identifying as White increased your likelihood of receiving home care [62, 71, 75, 104]. One study presents a more complex relationship: White Americans were more likely to receive any home care than African or Hispanic Americans, but Hispanics received the most home nursing and home health assistant visits per week [104].

##### Self-assessed health

Most studies found that there is an association between poorer self-assessed health and higher propensity of home care. Intensity of home care was not associated with poorer self-rated health as two of three studies found a null association [54, 78].

##### Insurance

Nine studies addressed the effect of an older adult’s insurance type on home care use. Insurance type is highly context dependent and six studies were conducted in the USA [44, 62, 65, 76, 77, 104], and one each in Ireland [85], China [79] and Singapore [101]. In general, having insurance played a positive role in receiving home care but commenting on type of insurance is not possible as comparison groups across studies are dissimilar. To illustrate, older Americans who were dually eligible for both Medicare and Medicaid were more likely to receive home care than those who were not eligible [44, 62]. In contrast, in the US, having private insurance was associated with increased home care use compared to older adults with only Medicare coverage [65].

##### Personal finances

The influence of personal finances, measured using varied income markers, is also uncertain. Three studies in Australia and Spain showed that receiving home care was associated with lower income [81, 83, 87]. On the other hand, a Chinese study found that higher income was associated with higher use of home care [78]. The uncertain association between personal finances and home care may reflect the differences in home care systems which have varied financial barriers, government subsidies and eligibility criteria for receiving home care [41]. One Canadian study observed that since home care eligibility is often means tested, combined with a robust Canadian social security system and pension plan, looking only at markers of income is insufficient to explain variations in home care use [75].

##### Housing Type & Problems

The care recipient’s home ownership status was considered in five studies [40, 58–60, 94]. There is an association between not owning your own home (i.e. renting or living in congregate housing) and increased propensity of home care use. The effect of home ownership on home care intensity is uncertain with two studies suggesting conflicting patterns of use. Older adults who lived in houses with either environmental hazards (housing problems) or lower numbers of rooms were associated with higher propensity of home care [42, 101].

##### Personality (psychosocial)

Older adults open to experiences (described as a tendency to be drawn to novel ideas, feelings, values, actions, and sensations) was positively associated with receipt of home care [61], as was being afraid to fall and thus choosing to restrict activities [57]. No association was found for older adults who expressed positive affect, purpose in life or fear of emergency situations [55, 59].

##### Other

Loneliness [49, 55], internet use frequency [49, 66], being born outside the country of study [57, 69, 83, 94] and engaging in certain health behaviours (smoking, drinking alcohol, seatbelt use and/or exercise) [44, 77, 103] did not demonstrate consistent association with home care use. Having good knowledge about home care services [57] has not associated with use of home care. Retirement [79], speaking French as a primary language in Canada [42], and driving as main method of transportation [76] were negatively correlated with home care propensity but were only examined in a single study precluding synthesis (not shown in Fig. 3).

#### Level: relationship

##### Living arrangements

**Fig. 4:**
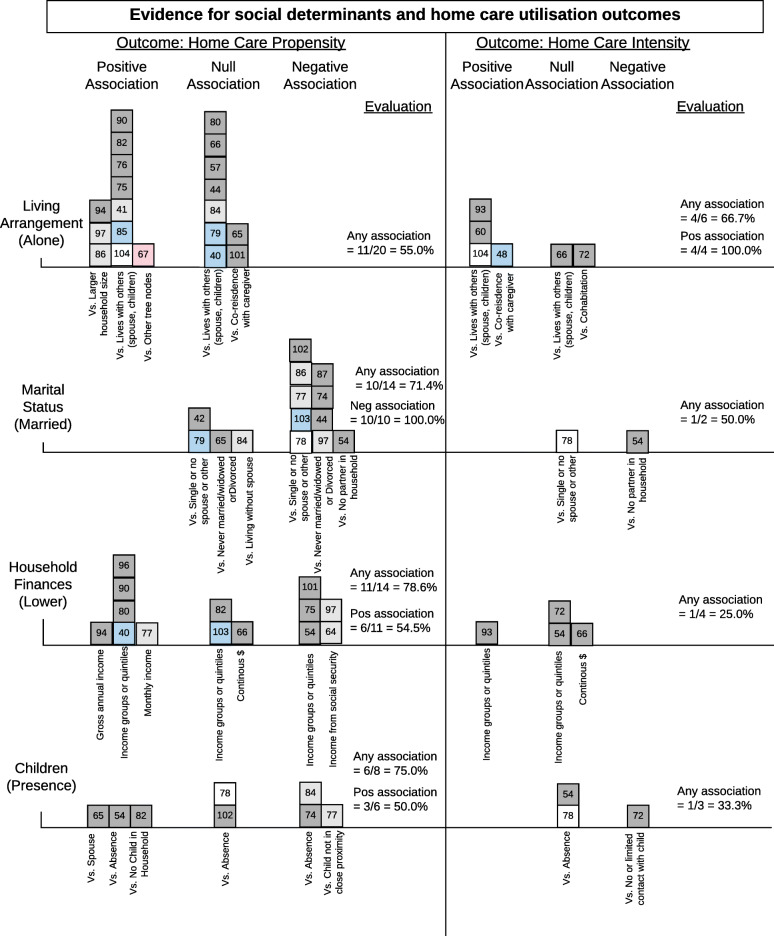
Harvest plot summarizing consistency of evidence for relationship level social determinants associatdc with home care propensity and intensity. Each social determinant is labelled on the left and each box represents one study. The number in the box corresponds to the study number in the references section. The social determinants are arranged in descending order based on counts of studies. The colour of the box indicates the study design (white = cohort, dark grey = secondary data analysis, light grey = secondary econometric analysis, dark blue = experimental, light blue = cross-sectional, pink = quality improvement). Where relevant, the x-axis is labelled with measures used to define the social determinant or to indicate a comparison group (denoted by vs.). The evaluation of aggregated findings is also summarized numerically in the right columns. HFSSM = Household food security survey module

The studies examining living arrangements showed an uncertain relationship with home care propensity and a significant positive association with home care intensity. While 11 studies suggested that living alone increased the likelihood of having received home care, nine other studies showed a null relationship. However, living alone was associated with greater amounts of home care (intensity) measured by cost [48, 60], time [93] and number of home health visits per week [104]. In Belgium, the total average formal support cost borne by the National Institute of Health and Disability Insurance (which covers 99% of the population) for older adults with IADL functional limitations was 725 EUR per month; the cost decreased with cohabitant carers and increased when living without a carer [48]. Details for relationship factors are summarized in Fig. 4.

##### Marital status

Older adults who were married were less likely to receive home care. One study found this association was especially strong for men [86]. Most noted that marriage is closely related to other social factors such as living arrangements and availability of any informal caregiving [54, 77, 78, 97, 102, 103]. Yet, after controlling for gender and the amount of informal caregiving received, Larsson and colleagues (2014) found that being married decreased the odds of receiving home help in the last five years of life by 62% but having children only decreased the odds by 40% [74].

##### Household finances

Across all studies, it appears that household finances were associated with home care propensity but the pattern of use is unclear. Six studies provided evidence that households with lower income received more home care [40, 77, 80, 90, 94, 96], but five studies suggested that higher income households received more [54, 64, 75, 97, 101]. Similar to personal finances, the influence of finances on home care is likely context and country dependent. One study analyzed data from a natural experiment whereby one cohort of low education Americans received a higher social security income than Americans born in a later cohort. They concluded that a $1000, or 10%, increase in annual Social Security household income decreased the likelihood of any nursing home use by 24–34% and increased the likelihood of receiving any paid home care use by 15–16% [64]. This result was echoed by Tsai (2015) who determined that home care was a normal good and was highly income sensitive [97]. There was no association between household income and home care intensity.

##### Children

While it appears that having children has an influence on home care propensity, the direction of the association is unclear. This uncertainty is further complicated as two studies compared receipt of home care related to types of children; older adults with sons were significantly more likely to receive home care than older adults with a daughter or daughter-in-law [96, 97]. Most studies suggested that the effect of children depends on their availability and willingness to provide informal care, but one study points out that national eligibility to access formal home care services in certain countries is contingent on children’s finances and proximity [84].

##### Informal Caregiving & Caregiver Burden

The availability of informal care decreased the propensity of home care but had no association with the intensity of home care received. This is a well-documented pattern, such that, “even in countries with extensive formal care supply, the majority of care is still informal and plays a major role in the support of older populations” [41] (pg.501). As such, when older adults doubted the availability of future informal care, there was a lower probability of using home care and a higher probability of using institutionalized care in one study [55].

Caregiver burden, measured by caregiver distress [42], caregiver capacity [65], family caregiver stress [54], the caregiver burden inventory [88] and caregiver burden score [80], did not show a significant association with home care across all studies. Of the five studies, only one demonstrated that higher caregiver burden was related to higher home care drop out rates [80]. A variety of caregiver characteristics were analyzed in relation to home care. These include caregiver education [65], health status [47, 55], employment [65, 84, 101], race [65] and transitions of roles [83]; no patterns of home care use emerged (results not shown in Fig. 4). Multiple studies suggested mechanisms by which informal care interacts with formal home care; informal care as complementary or substitutive for formal home care services was debated.

##### Social network

Five studies analyzed social networks in relation to home care, and each used a different measure [57, 65, 72, 75, 87]. Nonetheless, having a strong social network in which to rely on was associated with a lower likelihood of receiving home care.

##### Food insecurity

No independent association was noted between food insecure households and home care use.

#### Level: community

**Fig. 5:**
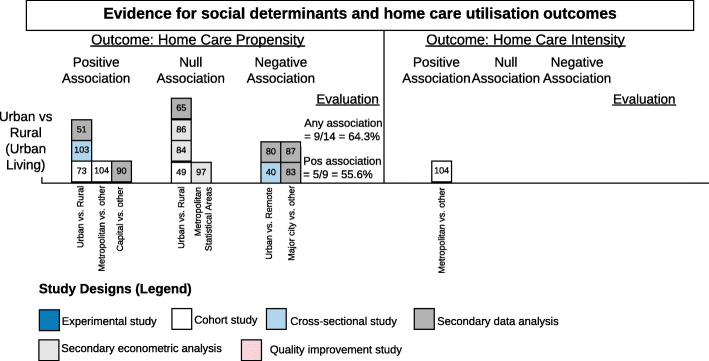
Harvest plot summarizing consistency of evidence for urban versus rural living and its association with home care propensity and intensity. Each box represents one study and the number in the box corresponds to the study number in the references section. The colour of the box indicates the study design (white = cohort, dark grey = secondary data analysis, light grey = secondary econometric analysis, light blue = cross-sectional). The x-axis is labelled with measures used to define the social determinant or to indicate a comparison group (denoted by vs.). The evaluation of aggregated findings is also summarized numerically in the right columns

Studies that analyzed community size frequently used an urban versus rural designation (Fig. 5). While more than 60% of the 14 studies show consistent evidence of an association with urban living and home care, the direction of the effect is equivocal. Six studies suggested living in an urban area is associated with higher receipt of home care [51, 73, 90, 103, 104] and five studies suggested the opposite [40, 80, 83, 87]. Understanding home care services based on rurality requires a more comprehensive view of other social, economic and political factors (across other levels of influence) that may be in play in each study. Several barriers to home care propensity in rural areas were described: infrastructure and transportation may be lacking [40, 51], home care budgets are poorer than in cities or services cost more due to travel times [84], and closure of primary care clinics that act as gatekeepers limits access to home care [51]. One study did show that rural older adults with home care were significantly less likely to stop using services compared to urban counterparts [80].

Communities of older adults were also evaluated based on geographical region but no patterns emerged across studies [40, 44, 66, 84, 97, 104]. Additionally, one study investigated communities based on composition of distinctive racial populations, but this did not appear to influence home care use [71]. Though, in a different study, neighbourhood problems (heavy crime, abandoned building, trash and litter) was related to higher probability of receiving home care [76].

The only randomized controlled trial identified by this review compared the effect of a multicomponent complex intervention that introduced new procedures for communication and follow-up using checklists within community home care services at defined stages in the patient trajectory [92]. Results of the study, including home care utilization, did not differ at 6 months post intervention. Two other community interventions were retrospectively evaluated and both were associated with increased home care propensity and intensity in their intervention groups compared with control groups. One implemented home-based primary care services and the other trialled a home care referral system made by paramedics [53, 99].

#### Level: societal

Societal level social determinants are related to the ways a society has chosen to govern their home care system. This includes methods of financing home care services, resource allocation and organization, structuring the delivery of these services and determining eligibility criteria, among others. Societal determinants also reflect prevalent norms, which “can be reflected through formal legislation as well as growing consensus of beliefs and homogeneity of values which pervade the society, thus shaping the health service system and utilization patterns” [106](p.9).

##### Payment for home care

Two studies analyzed the relationship between payment scheme for home care and the use of home care. In an American study, the odds of starting home health were higher for those enrolled in fee-for-service Medicare programs than the capitated Medicare Advantage programs, after adjusting for care recipient needs, demographics and socioeconomic factors [100]. Soga and colleagues (2020) also adjusted for care needs and found that increasing co-payment rates were associated with reductions in monthly LTC insurance charges (indicating a reduction in home care amounts) in two cities in Japan [95].

##### Supply and demand

Markets with higher competition levels and availability of complementary services (i.e. nursing home care, domestic cleaning services and day care services) predicted higher utilization of home care in the Netherlands [98]. Two studies determined that home care exhibits properties of a normal good (the quantity of home care demanded is expected to increase when income increases) [91, 97].

Two studies examined organization of home care services, both in Japan. Clients under private care agencies received higher home care propensity than those under public agencies, but public agency clients had higher intensity [105]. In contrast, no difference was found for care management agency ownership; however, monthly home care expenditures did increase if the agency delivering the services was part of the same agency case managing the services, as opposed to having two separate organizations for these roles [60].

##### Culture

At a national level, countries with stronger need based entitlements (especially the Netherlands and France) resulted in higher incidence rates of home care [63]. Marcinkowska and Sowa (2011) also suggest that countries with a Scandinavian approach were associated with higher levels of state responsibility (and robust social security programmes) and formal care services, overall. They conclude that there are undeniable differences in long-term care service patterns across the European Union (EU) due to traditions and social protection models; moreover, national home care regulations significantly influence provision of long-term care even after controlling for health, care needs and individual level social factors. Additional cultural norms that were identified were whether the country has universal entitlement, public financing and risk pooling, a home care system organized as part of health care as opposed to social care, and a culture of familialism [41, 43, 54, 63, 82]. Familialism was a term describing welfare states where families are presumed to take responsibility for their care recipients, rather than the state. This concept is closely related to norms of filial obligations, which directly influence social determinants from other levels of influence such as community resources, living situation, availability of informal caregivers and household composition [63].

A summary of all 35 social determinants across all four levels of the socio-ecological model is available in Table 2.

**Table 2:** Summary of all social determinants and their associations and patterns of home care use

Social Determinant (Descriptor)	Home Care Propensity Evaluation (Associated, Uncertain, Not Associated)	If Associated, Pattern of Home Care Utilization (More, Equivocal, Less)	Home Care Intensity Evaluation (Associated, Uncertain, Not Associated)	If Associated, Pattern of Home Care Utilization (More, Equivocal, Less)
Level in Socio-Ecological Model - Individual				
Age (Older)	Associated	More	Associated	More
Gender (Women)	Uncertain	–	Not Associated	–
Education (Higher)	Not associated	–	Associated	More
Ethnicity/ Race (White)	Associated	More	□	□
Self Assessed Health (Worse)	Associated	More	Not Associated	–
Insurance	Associated	H	□	□
Personal Finances (Lower)	Uncertain	–	Associated	Equivocal
Immigration (Born in Country of Study)	Uncertain	–	□	□
Housing Type (Own)	Associated	Less	Associated	Equivocal
Housing Problems (Present)	Associated	More	□	□
Internet Use (High Frequency)	Not Associated	–	Uncertain	–
Health Behaviours (Smoking, Alcohol, Seatbelts, Exercise)	Not Associated	–	□	□
Loneliness	Not Associated	–	□	□
Employment (Retired)	□	□	□	□
Language (French Speaking)	□	□	□	□
Transportation (Driving)	□	□	□	□
Knowledge (Home Care)	□	□	□	□
Level in Socio-Ecological Model - Relationships				
Living Arrangements (Alone)	Uncertain	–	Associated	More
Marital Status (Married)	Associated	Less	Uncertain	–
Household Finances (Lower)	Associated	Equivocal	Not Associated	–
Children (Presence)	Associated	Equivocal	Not Associated	–
Informal Caregiving (Available)	Associated	Less	Not Associated	–
Other Family Members	Not Associated	–	Uncertain	–
Caregiver Burden (Higher)	Not Associated	–	□	□
Social Network (Strong)	Associated	Less	□	□
Food Security (Insecure)	Uncertain	–	□	□
Level in Socio-Ecological Model – Community				
Urban vs. Rural (Urban Living)	Associated	Equivocal	□	□
Region	H	H	H	H
Community Programs	H	H	H	H
Racial Composition	□	□	□	□
Neighbourhood Problems	□	□	□	□
Level in Socio-Ecological Model - Society				
Payment Scheme for Home Care	H	H	H	H
Supply and Demand Factors	H	H	H	H
Organization of Home Care Services	H	H	H	H
Culture	H	H	H	H

Notes: Inadequate number of studies to perform evaluation of consistency across studies = □

Measures of social factors or comparison groups too heterogeneous for evaluations across all studies (see results section for thematic synthesis) = H

## Discussion

### Main Findings & How They Compare with Other Reviews

By providing an overview of social factors influencing home care utilization, this paper has brought together a wider range of social determinants in relation to home care utilization than those included in previous literature reviews. As summarized in Tables 2, 35 discrete social determinants were identified. This review also distinguished the level of influence at which these social factors were studied. Most studied social factors were properties of individuals or related to family units or peer groups. Social factors influencing home care were also identified at community levels (e.g. neighbourhood racial composition) and at societal levels (e.g. comparing national entitlement to home care systems), but these were less common.

There was a high degree of heterogeneity in the studies reviewed. Despite the differences with respect to patients sampled, countries of study, methods and analyses used, there were some findings that were similar across all studies. The factors consistently showing any association (positive, negative or equivocal in pattern) with home care propensity were: age, ethnicity/race, self-assessed health, insurance, housing ownership, housing problems, marital status, household income, children, informal caregiving, social networks and urban/rural area. Across all studies, social factors consistently showing any association (positive, negative or equivocal in pattern) with home care intensity were: age, personal finances, housing ownership and living arrangements.

For the social factors in commonality with previous reviews, these findings also support the findings of Kadushin (2004) and Johnson and colleagues (2018). The former concluded that living alone, a low level of informal support and Medicaid coverage have a relationship with higher use of home care services; it also noted most studies were from the USA [17]. The latter, a Canadian review, found that age, gender and location of residence influenced home care utilization [19]. Together, these reviews do not invalidate the message from other literature that links health status to the utilization of home care. Rather, independent of health-related needs, the reviews add to collective evidence supporting that social determinants influence home care utilization. In comparison to a review of formal community care in persons with dementia, not all factors were the same [18]. Ethnicity, living situation, region of residence and gender were compatible with this review but viewing formal care as a threat to independence and effect of previous experiences with home care were not. A number of explanations may explain these differences. By including qualitative studies, Bieber and colleagues (2019) were able to capture studies investigating attitudes towards home care and experiences with services (which were outside the scope of this review). Due to their population of interest (persons with dementia), the review by Bieber and colleagues (2019) strongly reflects the voice of the caregivers rather than the care recipients and it is well established that these two populations can have differing needs [18].

### Limitations

The findings of this review must be interpreted with caution. A number of limitations arose, especially the findings related to objective two (evaluating the influence of each discrete social factors on patterns of home care utilization). Generalising across studies is problematic given that 23 different countries and 64 data sources were included in the findings, each with different home care systems, cultural norms, study populations and designs, ways of measuring social factors, etc. For example, although one social factor (i.e. insurance) may be influential in one setting (i.e. USA), that same social factor may not apply in another country with different context (e.g. Netherlands which provides universal home care). Seeing the same social determinants repeated across multiple studies gives some confidence that there is a persistent association between these factors and home care and raises additional research questions regarding why these factors are consistent across many contexts; but each social factor is still best understood within its proper context. A key difference between many countries is the extent to which home care is organized. For example, nursing led versus social work led. In the United Kingdom (UK), it is social work led and nursing care is an entirely different service [49]. In Austria (part of SHARE), it is nursing led with a multi-disciplinary team involved in home care provision [82]. This would change the eligibility criteria for services, particularly as health systems tend to be universal in Europe, but social care services are often linked to social assistance programmes that are means-tested. In some countries like Germany there is also a further pillar – the long-term care system. As the social factors found by this review move from the individual to the societal levels in the model, the findings are increasingly variable. Even individual or relationship level factors that appear consistently associated across studies (i.e. house ownership or living arrangements) can be highly dependent on those variable community/societal factors (i.e. comprehensiveness of national social security programs). How these factors come together within a country to influence the direction of association is likely to be fairly unique.

Additionally, the method of evaluating outcomes across studies (see data synthesis section) was chosen as a well-documented way of comparing consistency across heterogeneous studies; this method has been used by other reviews on healthcare utilization [17, 21, 38]. However, this method produces results using a chain of procedural decisions. Evaluations using different tools or cut-offs may well lead to different findings. For social factors considered by small numbers of studies, this evaluation method may lead to erroneous conclusions.

Only studies in English were included in the inclusion criteria, again for feasibility reasons. Although this review found substantial numbers of included studies from Europe and Asia, additional literature written in non-English languages is likely missing. While this review followed rigorously defined scoping review methodology, a single reviewer conducted the screening and data extraction, which may potentially have introduced personal bias in these steps. Ideally, a double screening and extraction approach would have been carried out to increase internal validity and reliability, and reduce the chance of random error or personal bias. Since there was significant consultation between the authors to ensure adherence to the planned review protocol and to establish consensus for any difficult screening questions, it is unlikely that a double screening approach would change the overall findings of this paper.

Another important limitation of this review is the lack of quality appraisal of individual studies and grading of evidence for each outcome domain. Even though quality appraisal of studies in scoping reviews is controversial, its exclusion is often seen as a limitation to applying scoping reviews to policy and practice [26]. Only a quarter of scoping reviews published in 2014 included a quality appraisal step [18, 26]; hence, quality appraisal is not part of the traditional scoping methodology [25]. The lack of assessment of bias and lack of review of methodological quality may explain some of the inconsistencies in the results section. An assessment of methodological quality could be seen as especially important because most of the study designs included in this review were observational studies or secondary analyses; there was a noticeable absence of the traditional higher levels of evidence (i.e. RCTs).

In addition, the scope of this research question looked at home care use, rather than need or access. Access to, or need for, home care is a much more difficult concept to define and measure and would merit further research in this area. Ideally, use of home care correlates with need for care, but it is likely that this review fails to capture populations of older adults who lack the means to access home care altogether; hence a more vulnerable older adult population who could be adversely affected by social determinants of health.

### Policy implications

Overall, from this review, the take home messages for policymakers are clear: 1. Social factors influence home care use and should be accounted for in policy decisions; 2. These social factors should be understood as interdependent and not isolated from one another; 3. Enhancing home care infrastructure for older adults is inadequate so long as social circumstances make it difficult for individuals to remain at home. Rather, a multi-level approach can help create social circumstances and policies that make it safe, attractive and economical for individuals to use homecare; ideally helping to promote individuals living in the comfort and familiarity of their own home, shifting site of care away from institutions while improving cost-efficiency of the healthcare system. It may seem attractive to focus on individual or caregiver factors, but intervening at societal level (i.e. reducing co-payments or changing eligibility criteria) has potential to make population level shifts towards using more home care services; and 4. For policymakers who are interested in examining home care from a holistic perspective, there is a substantial body of literature that exists looking at factors influencing home care from all levels of influence in the socio-ecological model.

### Future research

This review addresses questions about *what* evidence on social factors and home care utilization is available and *which* social factors appeared consistently in the literature (breadth of the topic). But this paper does not answer *why* and *how* these social factors influence home care patterns. Understanding mechanisms is required for future research in this area. Understanding the complex, causal pathways by which social circumstances impact home care utilization requires recognition that some social factors are more relevant to specific contexts than others. For researchers who wish to look into the social factors influencing home care utilization in their communities/countries, Additional File 3 has compiled examples of data sources from each included country that would be conducive for this purpose. The results of this scoping review can also provide the basis for conducting a more focused systematic review (e.g. to establish the quality of evidence or answer questions regarding the effectiveness of social interventions). Finally, whether these social factors are impacting home care need or access (rather than home care use) is an area where future research can build on the limitations of this review.

## Conclusion

This paper provides a broad overview of social factors associated with home care utilization in community-dwelling older adults in high-income countries. It confirms social factors do influence home care utilization and highlights the diversity of social factors that have been studied in the literature. This scoping review brings together a wider range of social determinants in relation to home care utilization than those included in previous literature reviews. It also attempts to evaluate the influence of each social factor across all studies, but recognizes that these findings are limited by the high degree of heterogeneity and context dependency. This paper also organises its findings into a socio-ecological model to present a way of understanding and framing home care utilization related to interrelated social determinants.

## Supplementary Information

**Additional file 1.** Final Search Strategy for Scoping Review.

**Additional file 2.** PRISMA-ScR Checklist.

**Additional file 3.** List & Counts of Data Sources Divided by Continent and Country.

**Additional file 4.** Summary Table of All Included Studies & Study Characteristics.

## Data Availability

Supporting data and materials used in this paper can be accessed online through various medical databases.

## Abbreviations

ADL: Activities of Daily Living
EU: European Union
HHC: Home Health Care
HIC: High-Income Countries
HSS: Home Support Services
IADL: Instrumental Activities of Daily Living
LMIC: Low-and-Middle-Income Countries
LTC: Long-Term Care
LTCF: Long-Term Care Facility
LTCH: Long-Term Care Home
RCT: Randomized Controlled Trial
SDH: Social Determinants of Health
SHARE: The Survey of Health, Ageing and Retirement in Europe
UK: United Kingdom
USA: United States of America
