# Supplementary material for: Social factors influencing utilization of home care in community-dwelling older adults: a scoping review
Source: BMC Geriatr. 2021 Feb 27;21:145. doi: 10.1186/s12877-021-02069-1 (PMC7912889; doi:10.1186/s12877-021-02069-1)
Supplement: Supplementary file 3 — Additional file 3. List & Counts of Data Sources Divided by Continent and Country. [file 12877_2021_2069_MOESM3_ESM.docx]

Social factors influencing utilization of home care in community-dwelling older adults: A scoping review

## Additional File 3: List & Counts of Data Sources Divided by Continent and Country

Jasmine C Mah^1,2,3^, Susan J Stevens^4,5^, Janice M Keefe^4^, Kenneth Rockwood^6^, Melissa K Andrew^6^

^1^ Department of Health Policy, London School of Economics and Political Sciences, London, United Kingdom

^2^ Faculty of Public Health and Policy, London School of Hygiene and Tropical Medicine, London, United Kingdom

^3^ Department of Medicine, Dalhousie University, Halifax, NS, Canada

^4^ Faculty of Family Studies and Gerontology, Mount Saint Vincent University, Halifax, NS, Canada

^5^ Continuing Care, Nova Scotia Health, Halifax, Nova Scotia

^6^ Division of Geriatric Medicine, Dalhousie University, Halifax, NS, Canada

Total number of studies: 64 (excludes the two experimental studies)

| **Continent, Country** | | **Data Sources** | **Number of Studies** |
| --- | --- | --- | --- |
| **North America** | | | |
|  | **Canada** (total unique data sources = 6) | | |
|  |  | Canadian Community Health Survey | 2 |
|  |  | Health administrative data from the Institute of Clinical Evaluative Sciences | 1 |
|  |  | InterRAI-HC and linked service utilization data | 1 |
|  |  | Toronto Emergency Medical Service database linked to Community Care Assess Centre database | 1 |
|  | **USA** (total unique data sources = 20) | | |
|  |  | Administrative files of an upper Midwest Health Plan | 1 |
|  |  | Assets and Health Dynamics Among the Oldest Old Longitudinal Study (AHEAD) | 1 |
|  |  | Detroit City Wide Needs Assessment of Older Adults | 1 |
|  |  | Longitudinal Study of aging (LSOA II) | 1 |
|  |  | Medical Expenditure Panel Survey | 1 |
|  |  | Datasets from the Centers for Medicare & Medicaid Services   - Medicare Beneficiary Summary File linked with Medicare Provider Analysis and Review Database & Area Health and Resource File (AHRF) - Medicare Research Identifiable Files - Medicare data for a state - Medicare Provider of Services (POS) File linked with Outcome and Assessment Information Set (OASIS) and Area Health and Research File (AHRF) - Datasets from the Centers for Medicare & Medicaid Services linked with the Georgia Advanced Performance Outcomes Measures Project 6 | 5 |
|  |  | Medicare Residential History File | 1 |
|  |  | Medicare Primary and Consumer-Directed Care Demonstration | 1 |
|  |  | Mount Sinai Visiting Doctors Program | 1 |
|  |  | National Health Interview Survey | 1 |
|  |  | National Long-Term Care Survey and Informal Caregiver Data | 1 |
|  |  | Pathways to Life Quality Dataset | 2 |
|  |  | Health and Retirement Study | 3 |
|  |  | Outcome and Assessment Information Set (OASIS) | 1 |
| **Europe** (total unique data sources = 20) | | |  |
|  | **Multiple EU Countries** | |  |
|  |  | Survey of Health, Ageing and Retirement in Europe (SHARE) | 7 |
|  | **Belgium** |  |  |
|  |  | InterRai HC and ad hoc questionnaire | 1 |
|  | **Denmark** | |  |
|  |  | Danish Longitudinal Study of Ageing | 1 |
|  | **France** |  |  |
|  |  | Handicaps Incapacities Dependence (HID) Survey | 1 |
|  |  | Data from one departmental council | 1 |
|  | **Germany** | |  |
|  |  | Original questionnaires | 1 |
|  | **Ireland** |  |  |
|  |  | Irish Longitudinal Study on Ageing | 1 |
|  | **Netherlands** | |  |
|  |  | Linked national databases (national hospital discharge register, long-term care expenses register and population register) | 1 |
|  |  | Linked national long-term care register with NIVEL-PCD | 1 |
|  |  | Statistics Netherlands and Statline | 1 |
|  | **Spain** |  |  |
|  |  | European Health Interview Surveys for Spain (EHSS) | 1 |
|  |  | Disabilities, Independent and Dependency Situations Survey (DIDSS) | 1 |
|  | **Sweden** |  |  |
|  |  | Statistics Sweden and the National Board of Health and Welfare | 1 |
|  |  | Longitudinal study in Stockholm | 1 |
|  | **Switzerland** | |  |
|  |  | Lausanne Cohort Lc65+ population based study | 1 |
|  | **UK** |  |  |
|  |  | Well-being Interventions for Social and Health Needs (WISH) | 1 |
| **Asia** |  |  |  |
|  | **China** (total unique data sources = 3) | | |
|  |  | Dataset from Yanpu and Pudong Districts of Shanghai | 1 |
|  |  | Shanghai Long Term Care Needs Assessment Questionnaire (SLTNAQ) | 1 |
|  |  | Social Survey of Older People in Urban China | 1 |
|  | **Japan** (total unique data sources = 5) | | |
|  |  | Comprehensive Survey of Living Conditions of the People on Health and Welfare | 1 |
|  |  | Data from the Institute of Gerontology at the University of Tokyo | 1 |
|  |  | Long-term care bills from one city | 1 |
|  |  | Long-term care Insurance claims data | 1 |
|  |  | Minimum Data Set - Home Care (MDS-HC) | 1 |
|  | **Singapore** (total unique data sources = 1) | | |
|  |  | Singapore Longitudinal Survey for Long-Term Care Use | 1 |
|  | **Taiwan** (total unique data sources = 3) | | |
|  |  | Long-Term Care database from a city | 1 |
|  |  | Long-Term Care Management Information System (LTC-CM) | 1 |
|  |  | National Health Interview Survey | 1 |
| **Australia** (total unique data sources = 6) | | | |
|  |  | Australian Institute of Health and Welfare GEN Aged Care Data and Australian Bureau of Statistics | 1 |
|  |  | Australian Longitudinal Study of Women's Health (ALSWH) | 2 |
|  |  | National Census linked with Department of Health and Ageing Aged and Community Care Management System, & Aged Care Assessment Program Minimum Dataset | 1 |
