# Supplementary material for: Social factors influencing utilization of home care in community-dwelling older adults: a scoping review
Source: BMC Geriatr. 2021 Feb 27;21:145. doi: 10.1186/s12877-021-02069-1 (PMC7912889; doi:10.1186/s12877-021-02069-1)
Supplement: Supplementary file 4 — Additional file 4. Summary Table of All Included Studies & Study Characteristics. [file 12877_2021_2069_MOESM4_ESM.docx]

Social factors influencing utilization of home care in community-dwelling older adults: A scoping review

**Additional File 4: Summary Table of All Included Studies & Study Characteristics**

Jasmine C Mah^1,2,3^, Susan J Stevens^4,5^, Janice M Keefe^4^, Kenneth Rockwood^6^, Melissa K Andrew^6^

^1^ Department of Health Policy, London School of Economics and Political Sciences, London, United Kingdom

^2^ Faculty of Public Health and Policy, London School of Hygiene and Tropical Medicine, London, United Kingdom

^3^ Department of Medicine, Dalhousie University, Halifax, NS, Canada

^4^ Faculty of Family Studies and Gerontology, Mount Saint Vincent University, Halifax, NS, Canada

^5^ Continuing Care, Nova Scotia Health, Halifax, Nova Scotia

^6^ Division of Geriatric Medicine, Dalhousie University, Halifax, NS, Canada

| **Ref No.** | **Study, Date**  **(Country)** | **Research Aim** | **Research Method (Data, Year)** | **Sample Population (Sample Size)** | **Social Determinant Measures *** | **Dependent Variable** | **Key Findings in Relation to Present Review** | **Statistical Analysis Method (Accounted for Health Status)** |
| --- | --- | --- | --- | --- | --- | --- | --- | --- |
| 42 | Armstrong et al., 2015  (Canada) | To examine regional variation in service provision and identify client characteristics associated with OT and PT services in the Ontario Home Care System | Secondary data analysis of an Ontario service utilization and administrative database  (RAI-HC, 2005-2010) | Home care clients in Ontario, Canada, age ≥ 65 years (n=299,262) | Age, Caregiver Burden, Gender, Home Environment, Marital Status, Spoken Language | Provision of OT or PT services under home care | Predictors of increased OT use were home environment hazard and caregiver burden. Predictors of increased PT use were married status and home environment hazard. Increasing age, and French language decreased PT use. | Logistic regression  (Yes) |
| 43 | Bakx et al., 2015  (Netherlands and Germany) | To explain international differences in LTC use by investigating how differences in public LTC insurance impacted household decisions | Secondary econometric analysis of the Survey of Health, Ageing and Retirement in Europe (SHARE), first and second wave (2004, 2006) | Individuals 50 years and older and their spouses (n=8,739) | National LTCI comprehensiveness and eligibility | Receipt of formal care (self reported) | The probability of using any formal care is much higher in the Netherlands than in Germany. These institutional differences translate into very different relationships between LTC use – formal and informal – and personal characteristics such as income. After adjusting for covariates, the findings confirm that institutional and system differences (in coverage and eligibility) account for more of the between-country difference than patient characteristics. | Multinomial probit model  (Yes) |
| 44 | Bhargava and Lee, 2016  (USA) | To examine the relationship between food insecurity and the utilization of four types of health care services | Secondary data analysis of the National Interview Survey (2011, 2012) | American civilian residents, non-institutionalized, aged ≥ 65 years (n=13,589) | Age, Education, Food Insecurity*, Gender, Health Behaviours, Income, Living Situation, Marital Status, Race, Region, Insurance, Self Reported Health | Home Care use | After controlling for all covariates, food insecurity did not have an association with use of home health care. Increased home care use was associated with increasing age, never married or widowed, poor to good self rated health (compared to excellent) and dual Medicare & Medicaid insurance coverage. Living in the West of the USA was associated with less home care use. | Multivariate binary logistic regression  (Yes) |
| 45 | Bhargava et al., 2012  (USA) | To examine the relationship between food insecurity and Medicare related healthcare expenditures in low income older adults enrolled in Medicare and meal programs | Secondary data analysis of the Georgia Advanced Performance Outcomes Measures Project 6 and Centers for Medicare and Medicaid Services (2008 -2009) | All persons, average age 76.9 years, who participated in the Older Americans Act Nutrition Program (n=903) | Food insecurity | Probability and amount of Medicare home care expenditure | Food insecure participants were less likely to spend any healthcare expenditure than food secure participants, especially home health. Also, the adjusted mean total of Medicare home care expenditures for food insecure individuals was $474 less than those for food secure individuals. | Logistic regression and generalized linear models  (Yes) |
| 46 | Cameron et al., 2010  (USA) | To evaluate the relationship between gender and medical use among a national sample of community dwelling adults aged 65+ years | Cohort study through the Health and Retirement Study (2002, 2004) | Community dwelling adults aged ≥ 65 years (n=9,164) | Gender | Self reported use of home healthcare services | Women initially appeared to use significantly more home healthcare than men. However, after accounting for demographic differences, living arrangements, health needs and economic profiles, females and males made similar use of home care services. | Multiple logistic regression  (Yes) |
| 47 | Carlin and David, 2019  (USA) | To study the effect of informal caregivers on post-acute care and recovery of Medicare patients | Secondary data analysis of administrative files of an upper Midwest Health Plan (2014-2016) | Managed Medicare enrolees, average age 75.7 years, with an inpatient admission (n=19,815) | Informal caregiver presence and characteristics | Number of home health care visits post discharge | Compared to no caregiver, individuals with caregivers at home had a reduced likelihood of receiving home health care. Compared with a 70-year-old healthy male caregiver (reference category), caregivers under 55 years old or in poorer health were associated with greater use of HHC services. | Multivariable logit regression  (Yes) |
| 48 | Ces et al., 2020  (Belgium) | To estimate the direct costs (medical and non-medical) of supporting disabled elderly persons in their home | Cross sectional study using InterRAI-HC instrument, an ad hoc questionnaire and admin data (2010 -2016) | Disabled elderly persons, ≥ 60 years, at home fulfilling frailty or need criteria (n=5,642) | Presence levels of informal carer | Costs of formal support | Formal support costs were always significantly lower in situations with a cohabitant carer. | Univariate generalized linear models (Yes) |
| 49 | Clarke et al., 2017  (UK) | To examine the relationship between frequent internet use and different types of health and social care resource use | Cohort study through the Well-being Interventions for Social and Health Needs - WISH – Study (2012) | Community-dwelling older adults aged ≥ 65 years from five general practices in two regions of southern England (n=454) | Age, Education, Frequent Internet Use*, Gender, Loneliness, Race, Site | Contact with paid or unpaid home help | Receipt of assistance (paid or unpaid) for washing, cooking and similar tasks was not associated with frequent internet use when controlling for all the covariates (OR 0.56, 95% CI 0.12 to 2.55). | Multivariate logistic regression  (Yes) |
| 50 | Cotter et al., 2011  (Australia) | To investigate the effectiveness of the Australian Government's aged care planning framework for Indigenous Australians | Secondary data analysis of the national Census, Department of Health and Ageing Aged and Community Care Management Information System, and Aged Care Assessment Program Minimum Dataset (2005-2006) | Community-dwelling individuals ≥ 50 years (n=1,668 with Indigenous status and n=130,338 non-Indigenous Australians) | Indigenous status, stratified by age | Usage of aged care packages in the community | Community Aged Care Package usage rates are much higher for Indigenous than non-Indigenous people (for aged 50-69 years: 13.5% to 0.7% and for aged 70+ years: 68.2% to 15.3%). Ratio of usage of aged care packages in the community in Indigenous peoples aged 70+ compared to non Indigenous Australians aged 70+ is 4.46. | None described  (No) |
| 51 | Crouch et al., 2018  (USA) | To examine whether service utilization in the last six months of life differs across gender and rurality | Secondary data analysis of the Medicare Research Identifiable Files (2013) | Fee-for-service Medicare beneficiaries, of which 90.69% were ≥ 65 years (n=39,508) | Age, Gender*, Insurance, Level of Rurality*, Region | Use of home health services | After adjustment for covariates, the odds of rural beneficiaries using home health services in the last 6 months of life were lower than urban (aOR 0.87; 95%CI 0.81-0.93) and female beneficiaries were more likely to use home health services (aOR 1.07 95% CI 1.02-1.13) than males. | Multinomial logistic regression  (Yes) |
| 52 | de Almeida Mello et al., 2020  (Belgium) | To identify persons who are likely to enter residential care based upon their needs and resource utilization | Quasi-experimental study within a larger study called Protocol -3 (2010-2016) | Older adults ≥ 65 years, living in the community meeting frailty or functional criteria (n=10,289) | Age, Gender | Case Mix Index derived from the interRAI HC instrument | Being female showed an inverse effect on Case Mix Index. Age was not associated with Case Mix Index. | Linear regression models  (Yes) |
| 53 | De Jonge et al., 2014  (USA) | To determine the effect of home-based primary care (HBPC) on Medicare costs and mortality in frail elders | Case control concurrent study using Medicare administrative data (2004-2008) | Older adults ≥ 65 years, enrolled in the MedStar Washington Hospital Center's HBPC program. Controls matched on age, sex and Medicare status were from 3 American states (n=2,883) | A home based and mobile primary care innovation that delivers medical and social services to elders with severe and disabling chronic illness | Use and Medicare costs of skilled home health services | Individuals who received HBPC had a higher rate of skilled home health use (63.4% vs. 39.6% p<0.001), higher home health costs (6579$ vs. 4170$ p<0.001). | Univariate analysis with chi square and t-tests  (No) |
| 54 | Deindl and Brandt, 2017  (Europe – 12 Countries) | How is the lack of children in the support network compensated in older age? What role does social policy play, and how is informal and formal support for childless older people linked? | Secondary data analysis of the SHARE, first and second wave (2004, 2006) | People with IADL limitations, aged ≥ 50 years (n=14,394) | Age, Education, Gender, National Social Services, Self Rated Health, Socioeconomic Status, Social Networks | Receipt and intensity (weekly hours) of formal care in home | Associated with increased receipt of formal care was increasing age, worse self-rated health, being childless and a higher income. There was no association with gender, or presence of siblings/parents. Associated with increased intensity of formal care was higher education and not having a partner in house. A more generous welfare state with higher social services was associated with more use of formal care but fewer hours on average of different support types. | Multinomial multilevel logit models and linear multilevel regression models  (Yes) |
| 55 | Dorin et al., 2014  (Germany) | To analyze correlations between need characteristics and service utilization in home care arrangements | Cross sectional survey developed in cooperation with the Bertelsmann Foundation and a major German health insurance company (2012) | Older adults with severe care needs requiring more than 180 minutes of daily support and assistance (n=1152) | Age, Caregiver Burden, Caregiver Characteristics, Fear of Medical Situations, Gender, Informal Care, Missing Contact with Others, Wait Times | Utilization of one or more LTC services | Increasing age (81 years and older) (OR 641, 95% CI 9.14-694517, p=0.0003), worse caregiver health and long wait times all correlate with higher odds of LTC service use. Doubts of the future availability of the primary caregiver to give informal support is interestingly correlated with lower odds of LTC service use. | Multivariate logistic regression  (Yes) |
| 56 | Du, 2012  (USA) | To study the relationship between informal care and formal medical care utilization. | Secondary econometric analysis of the Health and Retirement Study – HRS (2004) | Single individuals over 70 years of age with at least one living child (n=2317) | Informal Care | Use of paid home health care | The results indicate that informal care provides a substitute for nursing home care and hospital inpatient care, but it does not affect paid home health care, on average. | Bayesian regression models  (Yes) |
| 57 | Dupraz, Henchoz and Santos-Eggimann, 2020  (Switzerland) | To quantify the home care use trajectories followed by community-dwelling participants in a cohort study of older adults during a 6-year timeframe | Secondary data analysis of prospectively collected data from the Lausanna cohort Lc65+ population study (2012-2018) | Community dwelling individuals ≥ 65 years (n=2,155) | Age, Born Abroad, Fear, Finances, Knowledge, Living Arrangements, Social Network | Formal care utilization | A difficult financial situation at baseline had higher odds of becoming formal care users (OR 1.65, 95% CI 1.12–2.45). Age, country of birth, knowledge of home care organizations, risk of social isolation and living arrangement did not show significant association. | Logistic regression  (Yes) |
| 58 | Ewen et al., 2017  (USA) | To identify differences in health, wealth and resources among adults residing in three housing types | Cross sectional descriptive study from Wave 2 of the Pathways to Life Quality dataset (2003) | Adults aged ≥ 50 years living in a variety of housing arrangements in an upstate New York county (n=672) | Housing Type | Number of Home and Community Based Services (HCBS) used | Service poor residents received more HCBS. Community dwelling residents used the least HCBS. | ANOVA and chi square tests  (No) |
| 59 | Ewen et al., 2017  (USA) | To examine how demographics, health status, psychosocial factors, and residence type are associated with HCBS utilization | Secondary data analysis of the Pathways to Life Quality Study (2003) | Community dwelling county residents aged ≥ 60 years in an upstate New York county (n=663) | Age, Gender, Housing Type, Marital Status, Psychosocial Factors (Affect, Life Satisfaction, Purpose in Life) | Use of HCBS and number of HCBS | Participants who resided in service-poor housing, those who were older, and female used more HCBS. Participants with worse positive affect and those with higher perceived purpose in life used more HCBS. | Linear regression models  (Yes) |
| 60 | Feng et al., 2017  (Japan) | To examine the relationship between clients’ service expenditures and whether the home help and day care service agencies belonged to the same organization as the care management agency | A secondary analysis of cross-sectional data collected by the Institute of Gerontology at the University of Tokyo (2010) | Individuals greater than 65 years with care needs who used at least one type of LTCI in-home service (n=4,331) | Age, Agency Relationship with Care Management*, Family Type, Gender, Household, Income, Type of Care Management Agency Ownership* | Monthly home help services (HHS) expenditure submitted to the national insurer | Living alone and living in a condominium/apartment were associated with higher home help service expenditure. Clients whose service agencies and care management agencies belonged to the same organization had higher expenditures, even after adjusting for confounders. | Linear regression models  (Yes) |
| 61 | Friedman et al., 2013  (USA) | To examine whether the Big Five personality traits were associated with seven expensive acute and long-term care services | Secondary data analysis of data collected for a randomized controlled trial, the Medicare Primary and Consumer-Directed Care Demonstration (1998-2002) | Medicare beneficiaries aged ≥ 65 years with chronic disabilities and significant health care use, living in a western NY state and 11 counties in West Virginia and Ohio (n=1,074) | Personality (Neuroticism, Extraversion, Openness to Experience, Agreeableness and Conscientiousness) | Use and amount of any skilled home health care use or custodial home care use | Higher openness to experience was associated with a greater probability of any custodial home care use. The predicted probability of any custodial home care use was 30% higher for patients high in openness to experience. | Logistic regression  (Yes) |
| 71  62 | Gandhi et al., 2018  (USA) | To examine racial disparities in health services utilization in Hawaii among Medicare FFS beneficiaries | Secondary data analysis of Hawaii Medicare data (2012) | Hawaiian residents, aged ≥ 65 years, with Medicare (n=84,212) | Age, Gender, Insurance, Race* (White, Asian, Pacific Islander, Other), Residential Area | Home health agency admissions, dichotomised | Whites were more likely to utilize all types of services, including home health more than any other racial group. Older age, female gender, Oahu region, dual insurance eligibility were also associated with higher use of home health. | Multinomial logistic regression  (Yes) |
| 41 | Gannon and Davin, 2010  (Ireland and France) | To analyse the current use of home care and estimate the relationship between formal and informal care | Secondary econometric analysis of SHARE (2006-2007) | Europeans from France or Ireland, aged ≥ 65 years (n=1,624) | Age, Children, Country, Education, Finances, Gender, Household, Informal Care* | Receipt of formal care | Living in France and living in a single household are associated with a higher probability of having received more formal care. More informal care is associated with lower probability of having received formal care. | Multinomial probit model  (Yes) |
| 63 | Geerts and Van den Bosch, 2012  (Europe – 9 Countries) | To explore how long-term care systems, and in particular the incorporation of needs-based entitlements to care services or benefits,  influence formal and informal care | Secondary data analysis of the SHARE, first and second waves (2004, 2006) | Persons aged ≥ 65 years from 9 European countries (n=6,293) | Institutional and cultural characteristics of LTC arrangements in each country, especially need based entitlements | Receipt of formal care | The likelihood of becoming a formal user varied significantly between countries. Whilst rates of formal care utilization continue to differ considerably between European countries, formal care allocation practices are not very dissimilar across Northern and continental European welfare states, as all countries target older persons living alone and the most care-dependent older people. | Logistic regression (Yes) |
| 40 | Gilmour, 2018  (Canada) | To present a descriptive analysis of socioeconomic characteristics of households receiving formal care | Cross sectional survey using Canadian Community Health Survey -CCHS (2015/16) | Canadian population, aged ≥ 12 years, excluding Indigenous settlements, Canadian forces and institutionalized populations (n=7,232) | Dwelling Ownership, Household Education, Household Income, Household Type, Place of Residence, Province / Territory, Source of Income | Receipt of formal home care | Parents living with children aged 25 or older, one-person households, lower income households, households with less than postsecondary education, households where the main source of income was Old Age Security of Guaranteed Income Supplement and renting a home were significantly more likely to have received formal home care. Households in large population centres and Western provinces or territories were less likely to receive formal home care. | Weighted frequencies and cross tabulations  (No) |
| 64 | Goda, Golberstein and Grabowski, 2011  (USA) | To estimate the impact of income on the long-term care utilization of elderly Americans using a natural experiment that led otherwise similar retirees to receive significantly different Social Security payments based on their year of birth | Secondary econometric analysis using data from the Assets and Health Dynamics among the Oldest Old – AHEAD -longitudinal survey (1993, 1995) | Community based elderly individuals born between 1901 and 1930, excluding those receiving below $100 per month. Analysis further focused on low education subsample (n=2,283) | Household Social Security Income | Paid home care use | Positive Social Security income shocks had a negative effect on nursing home entry, but a positive effect on the use of paid home care. Specifically, a $1000 (or 10%) increase in annual Social Security income for those in this low-education group increases the likelihood of receiving any paid home care use by 15–16%. | Probit models  (Yes) |
| 65 | Hong, 2010  (USA) | To identify patterns of service utilization among informal caregivers of frail older adults and examine determinants that explain heterogeneous variations in caregivers' service use patterns | Secondary data analysis of the National Long-Term Care Survey and Informal Caregiver data (2004) | Medicare beneficiaries aged ≥ 65 years with chronic disabilities living in the community and their primary informal caregivers (n=1908 dyads) | Age, Caregiver Characteristics*, Education, Family Income, Gender, Insurance, Marital Status, Residential Area | Patterns of formal care services (light service users, selective in-home service users and multiple service users) | Compared with light service users, care recipients using multiple services were more likely to be older, African American, rural living, educated and employed, have both private insurance and Medicaid and have higher levels of family income. Multiple service users were more likely to have caregivers that were spouses, had higher levels of social support, higher family cooperation over caregiving and higher caregiver burden. | Multinomial logistic regression  (Yes) |
| 66 | Hu et al., 2020  (China) | To investigate the characteristics of Chinese older people receiving home and community care and the factors associated with the sources of payment for care | Secondary data analysis of the Social Survey of Older People in Urban China (2017) | Older people, aged ≥ 60 years, in 10 large cities in different regions of China (n=3,247) | Age, Education, Gender, Living Arrangement, Number of Children, Online Activities, Perceived Proximity of Care, Receipt of Informal Care, Region of China, Self-Reported Need | Receipt of social care services | Perceived proximity of care is strongly associated with care receipt. For older people with services in the vicinity, their odds of receiving care are 18.8 times higher than the odds of older people without services in the vicinity. Females are more likely to receive care as do those with higher self-reported needs. Additionally, those who shop online have higher odds of receiving care but not other internet activities | Multilevel logistic regression and Poisson regression  (Yes) |
| 67 | Igarashi et al., 2014  (Japan) | To identify the patterns of combined community-based LTCI services used by older adults in need of care and the factors related to the selection of these service combinations | Quality improvement and evaluation project using the Minimum Data Set-Home Care - MDS-HC (2010) | All clients, aged ≥ 65 years or 40-64 years with specific age-related disease, with a specified care need level, assessed between Nov 2010 and Oct 2011 (n=983) | Living arrangements | Use of community-based LTCI services and combinations of LTCI community-based services | Combinations of services were related to living arrangements: older adults living alone had a home helper and those living with family used day care as the base of their care. This difference suggests that living arrangements impact service use, despite the government policy of providing appropriate services irrespective of family conditions. | Chi square autonomic interaction detection (CHAID) analysis  (No) |
| 68 | Ilinca, Rodrigues and Schmidt, 2017  (Europe – 12 countries) | What is the evidence of inequality, and horizontal inequity, by SES in the use of LTC at home across European countries? And which SES factors drive inequality and inequity in use of LTC in different European countries? | Secondary data analysis of the SHARE, fifth wave (2013) | Community dwelling older individuals aged ≥ 60 years, with LTC utilization and SES data (n=31,389) | Socio-economic status (SES) is proxied by equivalized net household income, obtained via the household level aggregation of all income components (including social benefits), equivalized using the square root scale and adjusted for purchasing power parity | Formal care services utilization | Most countries displayed a socioeconomic gradient in the use of home care services favouring the poor. However, after adjusting for higher needs in lower income groups, only Denmark and the Netherlands successfully target home care services to poorer individuals. Spain, a country that relies on means testing, has a clear pro-rich distribution of home care services. | Logistic regression (Yes) |
| 69 | Innes, 2020  (Sweden) | To study the likelihood of older migrants born in different countries using any kind of LTCS compared with the use by Swedish born older person | Secondary data analysis of registers at Statistics Sweden and the National Board of Health and Welfare (2015) | Swedish nationals, aged ≥ 65 years, excluding asylum seekers (n=~2,000,000) | Age, Age of Migration*, Children, Country of Birth*, Educational level, Income groups, Living arrangements, | LTCS (all, personal & domestic care) | When controlling for age at migration, there is great heterogeneity across and within the different birth countries, suggesting that later in life migration does not have to imply lower utilization. However, there are indications of a slight over-representation of personal and domestic care among older persons migrating after the age of 65. | Logistic regression (Yes) |
| 70 | Khadka et al., 2019  (Australia) | To determine age and gender specific incidence rates of aged care service utilization in Australia between 2008-09 and 2015–16 | Population based epidemiological study using data from the Australian Institute of Health and Welfare (AIHW) GEN Aged Care Data and the Australian Bureau of Statistics (ABS) (2008/9, 2015/6) | People ≥ 65 who were admitted to home care packages (HCP), residential aged care (RAC) and/or transition care (TC) (n=45,6916) | Age, Gender | Annual admissions into HCP, TC | Incidence rate of admission to HCP increased from 8.04/1000 in 2008-9 to 12.0/1000 in 2015-6 at an incidence rate ratio (IRR) of 1.52/year (p<0.001). The highest changes were seen in 80-89 year old males, whose access of HCP increased from 24.3/1000 to 37.9/1000 at an IRR of 1.65/year (p<0.001). Similar trends were seen for TC. | Poisson regression (No) |
| 71 | Kirby and Lau, 2010  (USA) | To investigate whether the interaction between individual race/ethnicity and community racial/ethnic composition is associated with health-related home care use among elderly persons | Secondary data analysis of Medical Expenditure Panel Survey and Decennial Census (2000-2006) | Community dwelling elders aged ≥ 65 years (n=23,792) | Race /Ethnicity (NH-White, NH-Blacks, NH-Asians or Hispanic) | Formal care receipt | No difference between NH-whites and NH-blacks or Hispanics was found; but the odds of having formal home care use for NH-Asian elders was only 25 percent that of NH-whites. Communities with >25% of one race did not show differences in formal care use, only informal care differences were observed. | Multivariate logistic regression (Yes) |
| 72 | Kjaer and Siren, 2019  (Denmark) | To examine individual level factors underpinning variations in care utilization | Secondary data analysis of the Danish Longitudinal Study of Ageing (1997, 2002, 2007/8, 2012/3) | Community-dwelling respondents, ≥67 years, who self-reported utilization of care (n=473) | Age, Contact with Children, Cohabitation, Gender, Having a confidant, Household income | Publically financed home-care service trajectory | Older age, no contact or limited contact with children, continuous cohabitating were more likely to be in the cluster of individuals who had a trajectory of increasing formal care utilization. | Multinomial logistic regression (Yes) |
| 73 | Kosar et al., 2020  (USA) | To describe rural-urban differences in post-acute care utilization and post discharge outcomes | Retrospective cohort study using Medicare Beneficiary Summary File, Medicare Provider Analysis and Review Database and Area Health Resource File | Medicare Advantage beneficiaries, ≥66 years, admitted to 4,738 acute care hospitals for stroke, hip fracture, COPD, CHF or pneumonia and tracked for 180 days post discharge (n=1,538,888) | County of residence (urban or rural or adjacent) | Discharge from hospital to community with home care | Patients from urban counties were more likely to be discharged to the community with home health care than rural patients. In comparison to patients in urban counties, patients in rural counties had longer home health episodes. | Multinomial logistic regression (Yes) |
| 74 | Larsson, Kareholt, and Thorslund, 2014  (Sweden) | To examine the effects of gender and marital status on the use of eldercare and hospital care in the last 5 years of life | Secondary data analysis from a longitudinal study in the inner city of Stockholm (1995-2004) | Individuals aged ≥75 years, registered in the parish of Kungsholmen in 1987 and were living either at home or in an institution (n=567) | Gender, Marital status, Parental status | Use of home help services | A higher proportion of unmarried than married people received home help. Among the unmarried, a higher proportion of women used home help. Living with a spouse decreased the odds of receiving home help by 62% and having children decreased the odds by 40% after controlling for gender. | Logistic regression (No) |
| 75 | Lee and Penning, 2019  (Canada) | To empirically assess to what extent individual-level indicators of social location, access to social and economic resources, and health related needs for care are associated with the receipt of informal care, formal care, or a mix of both informal and formal care among middle-aged and older adults with functional health limitations | Secondary data analysis from the Canadian Community Health Survey (2008-2009) | Persons aged ≥ 45 years (58.3% were ≥ 65 years) living in private dwellings in the 10 Canadian provinces (n=5,898) | Age, Education, Gender, Household income, Lives alone, Race, Social support | In-home care services (formal care and mixed formal and informal care) | Compared to no care, formal care use was higher in Whites. Compared to no care, formal care use was lower in younger age groups, those with less than a post secondary education, living with someone, and those reporting higher levels of social support. Compared to informal care, formal care use was lower in those reporting higher levels of social support, lowest incomes and younger age categories. Compared to informal care, formal care use was higher in men and Whites. | Multinomial logistic regression (Yes) |
| 76 | Lehning et al., 2013  (USA) | To examine the factors associated with urban African American elders’ utilization of home and community-based services and explore whether these factors differ by category of service | Secondary data analysis from the Detroit City Wide Needs Assessment of Older Adults (2001) | Non-institutionalized persons aged ≥ 60 years and African American (n=1,099) | Age, Education, Gender, Household income, Housing problems, Insurance, Living arrangement, Neighbourhood problems, Transportation | Use of any HCBS services (divided in 13 services and 5 categories) | Age, higher number of neighbourhood problems, Medicaid coverage and living alone had significantly increased odds of using any service use, while those who reported driving as their primary mode of transportation had decreased odds | Linear regression (Yes) |
| 77 | Li and Jensen, 2011  (USA) | To investigate the effects of privately purchased long-term care insurance  (LTCI) on three major types of long-term care services: nursing home care, paid home care, and informal care received from family and friends | Secondary econometric analysis of Health and Retirement Study (2002-2008) | Individuals aged ≥50 years who report they are limited in at least two out of five ADLs (n=6,551) | Age, Education, Gender, Income & Assets, LTCI*, Marital status | Paid home care use | Overall, LTCI’s effect on paid home care is small and statistically insignificant. However, for seniors who are moderately disabled (2-3 ADL limitations) private LTCI enables these older adults to avoid or at least postpone nursing home entry, making it possible for them to remain at home instead with paid home care. | Single equation maximum likelihood method (Yes) |
| 78 | Li et al., 2017  (China) | To examine the determinants of the use and expenses of formal care among in home elderly adults in Shanghai | Cohort survey embedded within the Shanghai Long Term Care Needs Assessment Questionnaire (SLTNAQ) (2014-2015) | Elderly aged ≥60 years living in the Jing'an district (n=7100) | Age, Children, Formal care fee paid, Gender, Living Arrangements, Marital status, Self reported health, Type of previous care | Probability of formal care use and formal care expenses | Older age, being females, those without a spouse, and those with a higher income had greater odds of formal care use. Use of formal care in 2014 was associated with use in 2015 but informal care in 2014 was inversely associated. Formal care expenses increased with age and income, but decreased if a formal care fee was required. | Logistic regression (Yes) |
| 79 | Liang, Liang and Corazzini, 2019  (China) | To explore the predictors and patterns of home health care among older adults in Shanghai,  China | A cross-sectional survey was conducted in the Yangpu and  Pudong Districts of Shanghai (2015) | Community dwelling adults over the age of 70 (n=829) | Age, Education, Employment, Gender, Income, Insurance, Living situation, Marital Status | Home health care receipt | Being retired had a negative association with skilled nursing care use (although less than 10% of the sample was still employed). Employee basic medical insurance had a positive association with skilled nursing care use. No other variables were found to have a significant association with skilled nursing care or chronic care home care. | Multivariable linear regression (Yes) |
| 80 | Liu et al., 2018  (Taiwan) | To examine the profile of home services utilization and the reasons for case closures, as well as the related influencing factors, among home service recipients in Taiwan | Secondary data analysis of the long-term care dataset of a major metropolitan area in Taiwan in the south (2011-2016) | Care recipients aged ≥ 50 years (average age 80.56 years) with dependency and home service assessments (n=7923) | Age, Caregiver burden, Gender, Household income, Literacy, Rurality, Subsidy gap, | Probability of leaving home services | Compared with care recipients from non-low income households, those from mid-low and low-income households were less likely to leave the service system. Higher caregiver burden was associated with greater risk of leaving home services (HR=1.041, p=0.003) | Cox regression (Yes) |
| 81 | Lopez-de-Andres et al., 2018  (Spain) | To describe the utilization of health and home care services among older people with diabetes during the economic crisis; to identify the factors associated with changes in the utilization of these services; and to study time trends | Cross-sectional studies using individualized data taken from the European Health Interview Surveys for  Spain (EHSS) (2009, 2014) | Non-institutionalized adults residing in main family dwellings in Spain aged ≥ 65 years (n=6,026 & n=6,020 for each year studied) | Age, Education, Gender, Income, Self-rated health | Self reported home care service receipt (HCS) | Older age, being a woman, lower income, fair/poor/very poor self-rated health, and those who did not practice physical activity in their free time exhibited significantly higher utilization frequencies of HCS. | Logistic regression (Yes) |
| 82 | Marcinkowska and Sowa, 2011  (Europe – 5 countries) | To identify patterns in the utilization of formal and informal long-term care across European countries and discuss possible determinants of demand for different types of care | Secondary data analysis of the Survey of Health, Ageing and Retirement in Europe (SHARE), second wave (2006) | Elderly persons aged ≥50 years (n=27,971) | Age, Children, Country Cluster in Europe, Education, Gender, Household income, Living Arrangements | Formal care receipt (public and OOP) | Overall, the highest volume of formal care provision is observed in the Netherlands, followed by Germany, Spain and Italy. After controlling for all personal characteristics, individuals are less likely to obtain formal LTC in Germany, Spain and Italy compared with the Netherlands. The results confirm that among the significant characteristics influencing the provision of LTC are the national regulations concerning the LTC system. | Multivariate analyses within country, and logit models for international comparisons (Yes) |
| 83 | McKenzie et al., 2014  (Australia) | To determine if changes in caring over time, including where carers live, influenced the relationship between the Andersen framework factors and home care service use | Secondary data analysis of the Australian Longitudinal Study on Women’s Health (ALSWH), second to fifth waves (1999-2008) | Women of the 1921-1926 cohort (n=21,363) | Age, Caregiver transitions*, Education, Country of birth, Income, Region | Use of three formal community support services | Women had higher odds of using nursing or community health services if they were older, lived outside a major city and reported difficulty managing on available income. | Generalized estimating equations with a predictor correlation structure (yes) |
| 84 | Mozhaeva, 2019  (Estonia) | To examine the association between socio-economic and need factors and utilization of informal and formal home care | Secondary econometric analysis of the Survey of Health, Ageing and Retirement in Europe (SHARE), second wave (2006) | Estonian elderly aged ≥60 years (n=4,108) | Age, Children, Education, Ethnicity, Gender, Household size, Household income, Living arrangement, Marital status, Region, Rurality, Self-assessed health Siblings alive | Formal care received | Increased use of formal care was associated with non-relative caregivers. Elderly persons with children were less likely to receive formal home care. | Ivreg2h model (yes) |
| 85 | Murphy, Whelan and Normand, 2015  (Ireland) | To provide a population-based estimate of the utilization of publicly financed formal home care by older adults in Ireland and to identify the principal characteristics of those utilising formal home care | Cross sectional design using data from the first wave of the Irish Longitudinal Study on Ageing (TILDA) | Participants aged ≥ 65 years living at a residential address in Ireland (n=3,507) | Age, Informal help, Insurance, Living arrangement, Self-rated health | Utilization of publicly financed home care services | Older adults (adjusted OR 3.4,  95% CI 2.4–4.8) were more likely than those in the younger age group to receive formal home care as were those living alone (aOR 2.6, 95% CI 1.9–3.8) compared to those living with a spouse/partner. For older adults without a disability, the most significant predictors of formal home-care utilization were increasing age (aOR 7.2, 95% CI 4.3–12.0), living alone (aOR 2.4, 95% CI 1.5–4.0) and receipt of informal help (aOR 3.0, 95% CI 1.9–4.8). | Multivariate logistic regression (Yes) |
| 86 | Paraponaris, Davin and Verger, 2012  (France) | To identify factors associated with the type of care (informal, formal, mixed or no care) received by the French disabled elderly and to assess relative costs | Secondary economic analysis of the Handicaps Incapacities Dependence - HID survey (1999) | Individuals who lived at home, aged ≥60 years, had a severe disability and needed help with ADLs (n=3,500) | Age, Children, Education, Gender, Household Size, Income, Rurality, Siblings | Receiving formal care | The probability of receiving formal care rises with age. The probability decreases among men, persons living with their spouse or someone else, persons having no degree, number of daughters or siblings and persons having more relatives. | Multinomial probit model (Yes) |
| 87 | Rahman, Efird and Byles, 2019  (Australia) | To identify patterns of aged care use among older Australian women and to examine how these patterns were associated with their demographic and health-related characteristics | Secondary data analysis of the Australian Longitudinal Study on Women’s Health (ALSWH), and administrative aged care and death datasets (2001-2011) | Women from the 1921-1926 birth cohort (n=8,768) | Income difficulty, Marital status, Region, Self-rated health, Social functioning score | Pattern of aged care use (non-user, basic HACC, moderate-high HACC/CACP, RAC) | Mostly moderate to high level HACC/CACP was associated with living outside a major city (OR=1.3, 95%CI=1.2–1.5), being widowed (OR=1.6, 95%CI=1.4–1.9), difficulty in managing income (OR=1.5,  95%CI=1.3–1.7). Furthermore, women with poor/fair self-rated health (OR=2.0, 95%CI=1.8–2.3) were more likely to be included in the mostly moderate to high-level HACC/CACP class than the mostly non-user class. | Latent class analysis (Yes) |
| 88 | Reckrey et al., 2013  (USA) | To examine associations between high caregiver burden and patient health care utilization in homebound elders | Longitudinal cohort study (2001-2002) | Patients (average age 79.5) from the Mount Sinai Visiting Doctors Program in New York who had an informal caregiver speaking English or Spanish (n=214) | Caregiver Burden Inventory dichotomized | Health care utilization (home visits and social work visits/calls) at 6 months follow up | There were no statistically significant associations between high caregiver burden and any utilization measure before and after adjusting for covariates | Multivariate logistic regression (Yes) |
| 89 | Rochon et al., 2014  (Canada) | To better understand how centenarians use the healthcare system as an important step towards improving their service delivery | Population based retrospective cohort study using linked health administrative data (2010) | All individuals living in Ontario aged ≥65 years on April 1 of each year between 1995-2010 (n=1,842 centenarians) | Age (grouped 100, 101-104, ≥105 years), Gender | Use of at least one publicly funded home care service in community | More males than females used home care (27.3 vs. 25%) despite female centenarians being more populous. In centenarians, younger age is associated with higher home care use (100 = 27%, 101-104 = 24.3% and ≥105 = 22%) | Descriptive statistics (No) |
| 90 | Rodriguez, 2013  (Spain) | To compare the explanatory factors for the use of each type of care among Spanish community dwelling dependent elderly | Secondary data analysis from the Disabilities, Independent and Dependency Situations Survey (DIDSS) (2007-2008) | Dependent older people, aged ≥65 living at home (n=10,703) | Age, Gender, Household income, Household size, Region, Self-rated health, | Receipt of formal care | Older age, and fair/bad/very bad self rated health and living in the capital associated with higher formal care use. Being male, living with a partner and higher income associated with decreased odds of receiving home care. | Multinomial logistic regression (Yes) |
| 91 | Roquebert and Tenand, 2016  (France) | To estimate the price elasticity of the demand for nonmedical home care services of the disabled elderly | Secondary econometric analysis from one departmental council in France (2012-2014) | Individuals ≥60 years, with a recognized disability and a needs of assistance, using an authorized home care provider and pays a co-payment of $0 or 90% (n=8,190) | Age, Gender, Income, Marital status, Price of home care* | Hours of home care | Overall, price elasticity estimates are negative and statistically significant (-0.387), confirming that the disabled elderly are sensitive to the price of professional home care. Also, home care is a necessity good for the disabled elderly. As expected, the highly disabled individuals consume relatively more (than the beneficiaries with mild to moderate disability) all other factors being equal. Even when controlling for the disability level, older age, being a woman, and living alone retain a significant effect on the consumption (increased) of formal home care services. | Tobit models (Yes) |
| 92 | Rosstad et al., 2017  (Norway) | To establish the effect of PaTH on patient level - compared to usual care - for elderly patients in need of home care services after discharge from a general hospital. | Un-blinded, cluster randomised controlled trial | Patients aged ≥70 years and served by one of the included home care clusters in Central Norway scheduled to receive home care services after discharge from hospital | PaTH, a multicomponent complex intervention that introduces new procedures for communication and follow-up using checklists within home care services at defined stages in the patient trajectory | Use of home care services at 6 and 12 months were secondary outcomes | The PaTH and control groups did not differ with respect to home care utilization at 6 months (OR 1.1 95% CI 0.7-1.8 p=0.62) or 12 months (OR 1.1 95% CI 0.7-1.8, p=0.60). | Logistic mixed models (yes) |
| 93 | Shih et al., 2020  (Taiwan) | To explore the utilization of formal home and community-based care under LTC policy  1.0 to add scientific support for the on-going LTC policy 2.0 reform | Secondary data analysis of the LTC-Care Management Information System (LTC-CM) (2013-2015) | Individuals eligible for LTC (n=101,457) | Age, Education, Family members, Gender, Living status, Region, Social welfare status | Length of time having received HCBS in the LTC system | The significant predictors of a shorter HCBS utilization period included being male, older, living in general service resources area and living in an area with a high proportion of certified nursing aids. Recipients who tended to stay longer in the LTC system included those living in lower income households, those who had a primary caregiver and those with higher BMIs. | Multiple regression analysis (Yes) |
| 94 | Slobbe et al., 2017  (Netherlands) | To develop a prediction model for long-term care using determinants collected from administrative data sources | Secondary data analysis of a large primary care database maintained by the Netherlands Institute for Health Services Research (NIVEL-PCD) linked to the national LTC-register and Statistics Netherlands (2006-2011) | Patients of GP practices registered for a continuous 3-year period, alive in 2012. Analyses conducted separately for those ≥ 65 years. | Age, Gender, Ethnicity, Household income, Household size, Household type (rent vs. home owner) | Long-term care utilization (divided into domestic, nursing, personal) | Older age, female, living alone, renting rather than owning a home are all associated with higher LTC home care of any type. | Multivariate logistic regression & generalized linear models (Yes) |
| 95 | Soga et al., 2020  (Japan) | To analyze the effects of raising insurance co-payment on patient-level LTCI claims data | Secondary analysis of anonymous LTC insurance claims dataset (2014-2016) | Individuals ≥65 years certified as requiring LTC in City A and City B, in Fukuoka Prefecture, Japan (n=7,711) | An increase in co-payment rate from 10% to 20% | LTCI service charges as a proxy for LTC costs | After adjusting for care needs levels and age, the co-payment rate increase was associated with reductions in monthly LTC insurance charges of $34.3 (P < 0.001) in City A and $91.0 (P = 0.022) in City B. | Difference-in-difference analysis and fixed effects models (Yes) |
| 96 | Tokunaga, Hashimoto and Tamiya, 2015  (Japan) | To examine the socioeconomic and demographic contexts of caregivers and their households | Secondary data analysis of the Comprehensive Survey of Living Conditions of the People on Health and Welfare (CSLCP) (2001, 2004, 2007, 2010) | Care recipient-caregiver dyads. The primary caregiver had to live within the same household and care recipients were aged ≥65 years (n=2,941 in 2001, n=2,725 in 2004, n=3,070 in 2007 and n=3,258 in 2010) | Caregiver characteristics, Household income | Visiting homecare services receipt | Caregivers who were never married/divorced sons or husbands were significantly more likely to use visiting homecare services compared with daughters in law. Households with the lowest income quintiles exhibited lower odds of using homecare services across the years. | Logistic regression (Yes) |
| 97 | Tsai, 2015  (USA) | To identify the effect of Social Security income on the use of formal and informal home care by the elderly | Secondary econometric analysis using the Second Longitudinal Study of Aging (1994) | Civilian non-institutionalized individuals born between 1900 and 1925 with a household social security income more than $100 per month who are Medicare insured (n=6,836) | Age, Children, Education Gender, Household size, Marital status, Race, Region, Size of community, Social Security income at household level* | Use of formal home care | Use of formal home care is a normal good and is highly income sensitive. Specifically, a $1000 increase in household Social Security Income would significantly increase the likelihood of utilizing formal care by 2.1 percentage points. Also, being male, being married and increasing size of household have negative impact on probability of formal home care. | Multinomial logit model with two-stage residual inclusion (No) |
| 98 | Van Noort et al., 2018  (Netherlands) | To explain home care costs in terms of demand and supply factors | Econometric analysis of linked databases from Statistics Netherlands and Statline (2015) | A convenience sample of 60 out of 408 municipalities in the Netherlands with participants who were receiving contracted care from health insurance company Menzis. | Age, Education, Income, Informal care, Rurality, Supply factors* | The % of inhabitants using home care services and the average annual costs per | Predictors of higher home care utilization are home care organizations that are integrated with intramural nursing homes, higher competition levels among home care organizations and the availability of complementary services. These supply factors explain 17-23% of variation in home care utilization | Multiple regression (Yes) |
| 99 | Verma et al., 2018  (Canada) | To assess the association between paramedic initiated home care referrals in Toronto and utilization of home care (and other services) | Retrospective cohort study using data from Toronto emergency medical services (EMS) linked to CCAC datasets (2011 - 2012) | Individuals who received home health referral by Toronto EMS paramedics, excluding those hospitalized, admitted to nursing home or died within 6 months. Mean age was 79.0 (n=1,851) | Community referrals by EMS program | Total combined hours of home services provided | EMS referral was associated with a significant increase in utilization of home care services. The proportion of the overall population receiving home care services increased from 18.2% to 42.5% after referral. There was an increase of 17.4 hours in total services per person in the six months after EMS referral (95% CI: 1.7–33.1, p = 0.03) | Nonparametric longitudinal regression (No) |
| 100 | Waxman et al., 2016  (USA) | To compare home health utilization and clinical outcomes between Medicare beneficiaries in the fee-for-service and Medicare Advantage programs | Secondary data analysis using the Outcome and Assessment Information Set (OASIS) and linked databases (2010, 2011) | Medicare beneficiaries with home health episodes, excluding dual beneficiaries of Medicare and Medicaid, average ages ranged from 76.96 (MA) to 77.66 (FFS) years (n=30,837,130 FFS and 10,594,658 MA) | Payment system (MA program versus FFS) | Initiation of home health and mean number of days enrolled in home health | The odds of starting home health during 2010 were 1.82 fold higher for those enrolled in FFS versus MA, after adjusting for patient characteristics and SES indicators. Among those who did start home health in 2010, FFS patients were enrolled an average of 34% more days than their MA counterparts, after risk adjustment | Logistic regression (Yes) |
| 101 | Wee et al., 2014  (Singapore) | To understand how formal CBLTC can complement family care | Secondary data analysis of the Singapore Longitudinal Survey for LTC use (2011-2013) | National referral database of individuals who decided to use CBLTC services (n=1,416) | Age, Caregiver characteristics, Education, Family members, Household income, Household rooms, Insurance | Use of Referred Community LTC Services (home-based) | Factors associated with less home- based CBLTC utilizations were: more rooms in house, older age of caregiver, and caregiver not working. Higher household income associated with more home based CBLTC use. | Logistic regression (Yes) |
| 102 | Wong et al., 2010  (Netherlands) | To investigate and compare predictors for hospital discharge of Dutch patients aged 65+ to alternative types of long-term care | Secondary data analysis of three linked national databases: the national hospital discharge register, the long-term care expenses register and the population register (2005) | Long-term care users aged ≥65 years, who were living at home prior to their admission to hospital and not utilizing any kind of formal care at the time (n=262,439) | Age, Caregiver characteristics, Children, Gender, Marital status | Discharge from hospital with home care | The presence of a spouse lowers the probability of home care at discharge compared to individuals with no spouse. Female care recipients and female caregivers were both more likely to use home care services, as were adults of older age until 90 years. After this age, the probability of home care utilization declined, and the probability of residential care increased. | Multinomial logistic regression (Yes) |
| 103 | Wu et al., 2014  (Taiwan) | To investigate the determinants of long-term care use and to clarify differences in the characteristics of home/community based and institution-based service users | Cross-sectional study using data from the National Health Interview Survey (2005) | Individuals aged ≥65 years (n=2,608) | Age, Education, Gender, Family members, Health behaviours, Household income, Marital status, Rurality | Long-term care services, public or private | Compared with users of institution-based care, users of home/community-based care were older, had significantly higher education, were more likely to be married or cohabitating and have more family members. For use of all LTC services compared with none, older age (OR=3.30, p=0.002), being single (OR =2.16) and living in an urban region (OR=1.68) were associated with higher use of any LTC service. | Logistic regression (Yes) |
| 104 | Yeboah-Korang, Kleppinger and Fortinsky, 2011  (USA) | To explore types and patterns of Medicare HHC services received by people with diabetes from different racial and ethnic backgrounds | Retrospective cohort design by linking Outcome and Assessment Information Set (OASIS), the Medicare Provider of Services (POS file) and Area Resource File (ARF) (2002) | Medicare beneficiaries with ≥ one complete episodes of HHC, with a primary home care diagnosis of type 2 diabetes mellitus who self reported as African American, Asian, Hispanic or white (n=9,838) | Age, Ethnicity/Race*, Gender, Insurance, Living arrangements, Region, Rurality | Receipt of HHC and visits per week, divided by type of service | For all HHC types, African Americans received fewer visits per week than whites, non-metropolitan residence was associated with fewer visits per week, living alone associated with more visits and geographic region. Being Hispanic, male, older in age, living alone, having a non-metropolitan residence and being Medicaid eligible conferred a greater likelihood of receiving HHA visits. | Multinomial logistic regression and multivariate linear regression (Yes) |
| 105 | Yoshioka et al., 2010  (Japan) | To compare differences in care management plans for community dwelling, frail, and elderly people between public care management agencies and private care management agencies | Cross sectional survey of City A LTC bills (2000) | City A west of Tokyo, users of in-home LTC service under the same care management agencies and whose care level remained unchanged throughout the 6 month period (n=309) | Care providing agencies: public sector, private sector, medical sector or other | Total LTC service use amount | In multivariate regression analysis, the utilization of community based long-term care service was significantly greater among beneficiaries managed by private agencies than those managed by public agencies  However, it is also suggested that the number of different services used is slightly higher in public agencies compared with private agencies | Multivariate regression analysis (Yes) |

Notes:

* - key social determinant examined in that study

Acronyms: ADL = Activities of daily living, CACP = Community aged care package programs, CBLTC = Community based long-term care, CCAC = Community Care Access Centers, CHF = Congestive heart failure, COPD = Chronic obstructive pulmonary disease, FFS = Fee-for-service, EMS =Emergency Medical Services, GP = General practitioner, HACC = Home and Community Care, HCBS = Home and Community Based Services, HCP = Home Care Packages, HCS = Home Care Services, HHA = Home Health Aides, HHC = Home Health Care, HHS = Home Help Services , IADL = Instrumental activities of daily living, interRAI-HC = The interRAI Home Care Instrument, IRR = Incidence risk ratio, LTC = Long-Term Care, LTCI = Long-Term Care Insurance, LTCS = Long-Term Care Services, MA = Medicare Advantage, OOP = Out-of-pocket, OT = Occupational Therapy , PT = Physiotherapy, RAC = Residential Aged Care, SES = Socioeconomic status, SHARE = Survey of Health, Ageing and Retirement in Europe, TC = Transition Care, UK = United Kingdom , USA = United States of America
